# Repurposing Metformin for Vascular Disease

**DOI:** 10.2174/0929867329666220729154615

**Published:** 2023-05-31

**Authors:** Chris R. Triggle, Isra Marei, Kevin Ye, Hong Ding, Todd J. Anderson, Morley D. Hollenberg, Michael A. Hill

**Affiliations:** 1 Department of Pharmacology & Medical Education, Weill Cornell Medicine in Qatar, PO Box 24144, Education City, Doha, Qatar;; 2 Department of Biomedical Physiology & Kinesiology, Simon Fraser University, Burnaby, British Columbia, Canada, V5A 1S6;; 3 Department of Cardiac Sciences and Libin Cardiovascular Institute, Cumming School of Medicine, University of Calgary, Calgary, Canada, T2N 4N1;; 4 Department of Physiology & Pharmacology, and Department of Medicine, Cumming School of Medicine, University of Calgary, Canada, T2N 4N1;; 5 Dalton Cardiovascular Research Center & Department of Medical Pharmacology & Physiology, School of Medicine, University of Missouri, Columbia 65211, Missouri, USA

**Keywords:** Metformin, vascular disease, type 2 diabetes, endothelium, inflammation, obesity, SARS-CoV-2, COVID-19

## Abstract

Metformin has been used as an oral anti-hyperglycaemic drug since the late 1950s; however, following the release in 1998 of the findings of the 20-year United Kingdom Prospective Diabetes Study (UKPDS), metformin use rapidly increased and today is the first-choice anti-hyperglycaemic drug for patients with type 2 diabetes (T2D). Metformin is in daily use by an estimated 150 million people worldwide. Historically, the benefits of metformin as an anti-diabetic and cardiovascular-protective drug have been linked to effects in the liver, where it acts to inhibit gluconeogenesis and lipogenesis, as well as reduce insulin resistance and enhance peripheral glucose utilization. However, direct protective effects on the endothelium and effects in the gut prior to metformin absorption are now recognized as important. In the gut, metformin modulates the glucagon-like peptide-1 (GLP-1) - gut-brain axis and impacts the intestinal microbiota. As the apparent number of putative tissue and cellular targets for metformin has increased, so has the interest in re-purposing metformin to treat other diseases that include polycystic ovary syndrome (PCOS), cancer, neurodegenerative diseases, and COVID-19. Metformin is also being investigated as an anti-ageing drug. Of particular interest is whether metformin provides the same level of vascular protection in individuals other than those with T2D, including obese individuals with metabolic syndrome, or in the setting of vascular thromboinflammation caused by SARS-CoV-2. In this review, we critically evaluate the literature to highlight clinical settings in which metformin might be therapeutically repurposed for the prevention and treatment of vascular disease.

## 1 INTRODUCTION

Metformin was first used for the clinical management of T2D in 1957 [1]. Despite being in use for over 60 years and subjected to intensive research, the mode of action of metformin as an anti-hyperglycemic drug remains controversial [2]. Although the primary clinical use of metformin is for T2D, it is used to treat polycystic ovary syndrome (PCOS) and has been investigated for type 1 diabetes [TID], cancer, neurodegenerative diseases, as an anti-aging drug, and also for the treatment of infectious diseases, including COVID-19 [2]. The common mediator of some, but not all, of the cellular actions of metformin has been attributed to 5’-adenosine monophosphate activated protein kinase (AMPK), and other activators of AMPK have been shown to have comparable effects to metformin in rodent models of hyperglycemia and hyperinsulinemia [3, 4].

## 2 METFORMIN AND THE UNITED KINGDOM PROSPECTIVE DIABETES STUDY (UKPDS)

The key conclusions from the United Kingdom Prospective Diabetes Study (UKPDS) were that controlling blood glucose levels reduced the complications of T2D in newly diagnosed patients and reduced mortality [5-7]. In addition, in a subgroup of 1704 subjects who were overweight (BMI ≥ 25 kg/m^2^), the relative risk reductions in those receiving metformin were significantly larger than those achieved in the overweight group receiving intensive treatment with sulfonylureas (either the first generation chlorpropramide, or second generation, glibenclamide), or insulin [6].

### 2.1 Confirmation of Conclusions of the UKPDS

The conclusions of UKPDS that specifically indicated a reduced cardiovascular (CV) morbidity and mortality with metformin monotherapy versus sulfonylurea monotherapy have been supported by a number of follow up analyses in patients with T2D. Data from a 10-year follow up in 2008 indicated that treatment with metformin was associated with a continued reduction in myocardial infarctions despite no change in HbA1c [8]. Complementary data have also been provided by other studies, including a retrospective analysis of Saskatchewan Health databases [9]. Expanding the evidence to diverse ethnic groups, comparable results have also been reported in a prospective study of 1157 subjects identified in the Taiwan Society of Cardiology registry, comparing those with T2D and acute coronary syndrome, where lower all-cause mortality was associated with treatment with metformin [10]. A post-hoc analysis of data from the SAVOR-TIMI 53 (Saxagliptin and Cardiovascular Outcomes in Patients With Type 2 Diabetes Mellitus) trial also showed that treatment with metformin in patients with T2D reduced the risk of all-cause mortality, although this study found no significant relationship between metformin use and a reduction in nonfatal stroke, nonfatal myocardial infarction, and cardiovascular death (3P- MACE, Major Cardiovascular Events) [11].

### 2.2 The Diabetes Prevention Program (DPP)

The results of the Diabetes Prevention Program (DPP) research group, although providing support for the weight loss and the safety benefits of metformin, concluded that the benefit of lifestyle intervention alone was greater than that resulting from metformin; and furthermore, the combination with metformin was less beneficial than lifestyle intervention alone [12, 13]. In addition, a meta-analysis provided information for over 1 million patients receiving treatment with metformin enrolled in 40 studies that reflected 15 randomized, 22 retrospective and 3 case-based studies also provided conflicting results [14]. The meta-analysis showed that although metformin effectively reduced all-cause mortality, cardiovascular mortality, and the incidence of cardiovascular events in patients with T2D, comparable positive results were not seen in patients without diabetes and with myocardial infarction (MI) or coronary artery disease (CAD). Moreover, treatment with metformin did not lower LDL levels [14]. Furthermore, based on another meta-analysis of 13 randomized trials (including data from the UKPDS) that included a total of 2079 overweight subjects with poor glycemic control and aged ≤65 years, a significant cardiovascular benefit to using metformin was not apparent, although outcomes favoured metformin [15].

Concerns over potential bias and the small number of patients in the metformin study in the original UKPDS analysis have also been raised by earlier meta- analysis [16, 17]. The conclusions from the 2012 meta- analysis involving over 9,000 patients in 13 studies were that because of the considerable heterogeneity in the data, with as much as a 33% reduction versus as high as a 64% increase in cardiovascular mortality, the risk/benefit ratio for the use of metformin could not be accurately estimated without additional evidence [16].

Collectively, these data indicate the need for new prospective studies that compare metformin with the newer anti-hyperglycemic agents, notably the GLP-RAs and SGLT-2 inhibitors.

## 3 METFORMIN AND ENDOTHELIAL FUNCTION

### 3.1 Endothelium and Vascular Health

The endothelium plays an essential role in maintaining a healthy cardiovascular system with endothelial cells functioning akin to an endocrine organ *via* their contribution of vasoactive molecules, low resistance coupling to the underlying vascular smooth muscle cells, enhancing vasodilation and blood flow, and suppressing pro-thrombotic events [18-20]. A direct *in vivo* assessment of the health of the endothelium can be accomplished by determining the effectiveness of an infusion of an endothelium-dependent vasodilator such as acetylcholine, using strain gauge plethysmography following, for example, forearm venous occlusion or determining flow-mediated vasodilation with ultrasound (FMD) [20]. Alternatively, or in combination with the measurement of vasodilation, measurements of biomarkers of vascular inflammation such as C-reactive protein (CRP), as well as P-selectin, vascular cell adhesion molecule-1 (VCAM-1), and intercellular adhesion molecule (ICAM)-1 can be used as surrogate measures [20]. Endothelial dysfunction is considered to be a *‘barometer’* for cardiovascular risk and can be defined as a reduction in endothelium-dependent vasodilation (EDV) in response to endothelium-dependent vasodilators such as acetylcholine [20, 21]. Endothelial dysfunction is an important and the earliest detectable indicator of the development of cardiovascular disease [20-23].

### 3.2 Metformin and Endothelial Function in Type 2 Diabetes

A substantial amount of clinical and pre-clinical data indicates that metformin has direct vascular effects on protecting and improving endothelial function. Recent reviews are also available [19, 26, 27]. That metformin improves endothelial function is supported by several clinical studies [28-30]. In the 2001 study by Mather *et al.*, 44 subjects with diet controlled metformin naïve, T2D, and fasting plasma glucose levels of > 7.0 mmol/L were treated for 3 months with metformin (500 mg/bid) or placebo following a design where patient identity and treatment were blinded. In these T2D patients, forearm blood flow was measured using strain-gauge plethysmography and the effects of the intra-brachial artery administration of the endothelium-dependent vasodilator, acetylcholine *versus* the effects of the endothelium-independent vasodilators, sodium nitroprusside, and verapamil. The results showed that metformin lowered whole body insulin resistance (assessed as HOMA-IR), improved acetylcholine-mediated EDV, without an affect on either sodium nitroprusside or verapamil-mediated, endothelium-independent, mechanisms. The authors concluded that treatment of patients with T2D corrected endothelial function secondary to the reduction of whole body improved insulin resistance [28]. Comparable results and conclusions have also been reported in patients with metabolic syndrome where treatment with metformin (500 mg bid) for 3 months improved FMD in the brachial artery [29]. Confirmation also comes from a larger randomized placebo-controlled study with 390 patients treated with metformin (850 mg/tid) and a 4.3 year follow up that demonstrated that metformin lowered the plasma levels of biomarkers of inflammation and endothelial dysfunction including CRP, C-reactive protein; von Willebrand factor, vWF; soluble vascular adhesion molecule-1, sVCAM-1; soluble E-selectin, sE-selectin; tissue type plasminogen activator, t-PA; and plasminogen activator inhibitor-1, PAI-1 [31]. However, concerns have been raised that the chronic use of metformin may result in vitamin B12 deficiency [27].

### 3.3 Metformin and Endothelial Function Independent of Anti-Hyperglycaemic Actions

In view of the positive data derived from both pre-clinical investigations as well as clinical studies in patients with type 2 diabetes, it might be expected that the beneficial effects of metformin on endothelial function should extend to subjects with vascular disease unrelated to diabetes. Evidence that metformin improved endothelial function was first reported in 1984 with data from a 6-month placebo driven randomized controlled study of the effects of 850 mg/day of metformin *versus* placebo on FMD in subjects who were non-diabetic but with peripheral artery disease [32]. Although there were no changes in fasting glucose or insulin levels, metformin improved FMD and the lipid profile [33]. A three-month treatment with metformin has also been reported to improve EDV mediated by acetylcholine in 31 first-degree relatives of T2D patients with metabolic syndrome but normal glucose tolerance [34]. The benefits of metformin on endothelial function also extend to subjects with T1D with the addition of 850 mg/tid (three times a day) of metformin to their standard insulin regimen for 6 months reported to improve FMD, but not endothelium-independent vasodilation mediated by infusing glyceryl trinitrate, and without changing the metabolic profile of the patients [35]. However, as detailed below, not all studies have produced positive data and a number of prospective studies designed to determine whether metformin has CV protective benefits independent of its anti-hyperglycaemic actions have been negative; see also Rena and Lang, 2018 [36].

The conclusion from a recent meta-analysis reported by Han *et al.* (2019) [14] was that although metformin effectively reduced all-cause mortality and cardiovascular mortality in patients with T2D, comparable positive results were not seen in patients without diabetes. Rena and Lang (2018) have summarized the results from a number of prospective trials, some still ongoing, that have investigated whether metformin has comparable benefits to those reported in T2D for subjects without diabetes [36]. On the positive side, in a randomized double-blinded placebo-controlled study, metformin treatment (500 mg/bid) for 8-weeks of 33 non-diabetic women with exercise-induced ischemia improved endothelium-dependent vasodilation (to acetylcholine) but not endothelium-independent vasodilation (to sodium nitroprusside) while also improving exercise tolerance [37]. On the other hand, in the CAMERA study (Carotid Atherosclerosis Metformin for Insulin Resistance trial: NCT01483560), metformin did not reduce coronary artery intima-media thickness (cIMT) as measured by ultrasound after 1.5 years of treatment of 173 non-diabetic subjects who were already receiving a statin [38, 39]. Metformin did, however, lower HbA1c and insulin resistance and tissue plasminogen activator, but not fasting glucose, CRP, or total cholesterol [38].

### 3.4 Cellular basis for a Direct Action of Metformin on Endothelial Function

The putative cellular pathways whereby metformin enhances endothelial function include a reduction in reactive oxygen species (ROS) in part through metformin’s anti-hyperglycaemic actions; activating AMPK with downstream effects on multiple cellular pathways; enhancing endothelial nitric oxide synthase (eNOS) activity *via* multiple mechanisms including reducing ROS, activation of AMPK, as well actions on microRNAs (miR34a), and also sirtuin-1. There is, however, no evidence that metformin directly relaxes vascular smooth muscle through altering calcium homeostasis, although the allosteric activator of AMPK, A-769662, has been reported to lower intracellular calcium levels in intact rabbit blood vessels [40]. Other studies have, however, demonstrated vasodilator/vasorelaxant effects of metformin in retinal arterioles and aortic tissue that is mediated *via* the activation of AMPK, albeit requiring high concentrations of metformin [41, 42]. The effects of metformin on endothelial and vascular function have been extensively reviewed and will not be discussed in detail; however, reference is made to several recent reviews [19, 24-27].

#### 3.4.1 Reactive Oxygen Species (ROS)

Oxidative stress associated with diabetes has been attributed to the hyperglycaemia-induced generation of ROS from mitochondria [43]. Thus, metformin by virtue of its anti-hyperglycaemic actions, will reduce the generation of ROS from mitochondria. Metformin has also been shown to reduce the generation of ROS in human umbilical vein endothelial cells (HUVECs) elicited by Advanced Glycation Endproducts (namely carboxymethyllysine and S100 proteins) and also palmitic acid [44]. Similar findings has been reported in rat aorta endothelial cells [45], as well as in the microvasculature [46] and has been linked to the activation of AMPK, increased eNOS phosphorylation, as well as a reduction in the activation of NF-κB [47].

#### 3.4.2 Sirtuin-1

*In vitro* data obtained using cultured mouse microvascular endothelial cells (MMECs) and silencing siRNAs indicate a critical role for sirtuin-1 in the signaling pathway by which metformin reduces oxidative stress and protects endothelial cells against hyperglycaemia-induced senescence [48]. Data from Zheng *et al.* (2012) suggest that the activity of sirtuin 1 in endothelial cells is directly enhanced by metformin [49]. Importantly sirtuin-1 is a NAD-dependent deacetylase and the protein product of the so-called ‘anti-aging’ gene with putative anti-aging benefits demonstrated in yeast, Caenorhabditis elegans, and Drosophila [50]. Sirtuin-1 expression is reduced by insulin resistance [51]. In addition to its protective effects against oxidative stress and senescence, sirtuin-1 also plays an essential role in the regulation of angiogenesis and positively regulates *via* deacetylation of the serine-threonine kinase, liver kinase B1 (LKB1) that is upstream of AMPK and argued to play a protective role against disease progression [52-55]. Furthermore, sirtuin-1 also deacetylates lysines 496 and 506 on endothelial nitric oxide synthase (eNOS), enhancing NO-mediated EDV [56]. Collectively these data suggest a potential mechanism that links the protective effects of metformin on endothelial cells and improved vascular function with the putative and controversial anti-aging effects of metformin, as has been recently critically reviewed [57].

#### 3.4.3 Nr4a1

*In vitro* studies with isolated murine blood vessels, endothelial cells in culture and *in silico* modeling have also identified the orphan nuclear receptor, Nr4a1 (Nur77), as its expression is critical for mediating the protective effects of metformin on endothelial function against the effects of hyperglycaemia [58]. Remarkably, the protective effects of metformin are observed in the low micromolar range, 1 to 10 μM, but protection is lost in endothelial cells and blood vessels from Nr4a1 null mice [45]. The effects of metformin also extend to protecting mitochondrial function without, except at high concentrations, inhibition of complex 1 and indicate the potential therapeutic significance of Nur77 in mediating the vascular protective effects of metformin in patients with T2D [58]. Of significance, Nur77 regulates the localization of LKB1 [59]; however, the signaling pathway that mediates the protective effects of metformin on endothelial cell function *via* Nur77 requires further investigation.

### 3.5 Metformin and Endothelial Progenitor Cells

The capacity of endothelial cells for self-renewal is limited, and endothelial progenitor cells (EPCs) that circulate in the blood play an important role in endothelial repair. It, therefore, follows that EPCs are targets for drugs, such as metformin that potentially can enhance their repair capacity. In 1997, Asahara *et al.* first described spindle shaped CD34+ EPCs from human blood that, similarly to mature endothelial cells, expressed eNOS, and the human homolog of VEGFR2 (KDR), differentiated into endothelial cells and were shown to be pro-angiogenic in a mouse hind limb ischemia model [60]. The discovery of circulating EPCs indicated that neovascularization does not necessarily depend on endothelial cells in the tissue. Subsequent to this 1997 publication, there has been considerable interest in the role of EPCs in diabetes and whether targeting and protecting EPCs from the diabetic environment may be a viable approach to offset the negative effects of diabetes on cardiovascular function. EPCs are believed to participate in vascular repair processes through their incorporation into the injured endothelium [61] and/or the paracrine activation of regenerative resident endothelial cells [62]. Other studies suggest that EPCs also have an autocrine function exerted through the regulation of signaling pathways such as those involving Akt, nuclear factor-kappa B, STAT, and Notch [63, 64].

To date, there is substantial variation in exactly how EPCs are defined, and several subpopulations have been classified under this general term [65]. Two main subpopulations of EPCs (early and late EPCs) have been described based on their isolation method (Table **1**) [65]. It is hypothesized that both subpopulations play synergistic roles in vascular repair [66]. Early EPCs are thought to be involved in vascular repair through the paracrine regulation of neovascularization [67, 68]. Late EPCs are thought to respond to the paracrine signals secreted by white blood cells (and early EPCs) and to incorporate into the site of injury [62].

Available data suggest the involvement of more than one subpopulation of EPCs in the pathogenesis of diabetes and its complications [69-74]. Studies on early EPCs demonstrated an increase in the number of senescent cells isolated from diabetic patients, and this was correlated to elevated levels of endogenous eNOS inhibitors (asymmetric dimethylarginine and dimethylarginine dimethylaminohydrolase) [72]. In addition, functional properties such as proliferation and migration have been shown to be impaired in EPCs isolated from patients with T2D [75]. It was shown that the multifactorial treatment of diabetic patients involving lifestyle changes together with glycemic, lipid, blood pressure and antithrombotic therapy enhanced the numbers of early EPCs [73]. Studies investigating the effects of hyperglycaemic conditions on late EPCs showed that exposing healthy late EPCs to elevated glucose levels resulted in impaired eNOS phosphorylation [76] and reduced the generation of NO [77].

Studies on progenitor cells isolated using antigen selection have shown that this approach is associated with reductions in cell yield and function of EPCs [69-71]. These studies rely on the selection of CD34+ cells combined with other markers (such as KDR, CD133, Sca-1, Flk-1, and CXCR4) [69-71]. Further, impaired mobilization of CD34+KDR+ EPCs in diabetic subjects was shown [69], as was a reduction of CD133+/KDR+ EPCs [70] and CD34+KDR+ EPCs [69, 71] in T2D and its association with peripheral vascular disease risk [69]. Furthermore, when healthy CD34^+^/ KDR+ EPCs are subjected to a hyperglycaemic cell culture protocol, both NO generation and cell migration are reduced [74].

Collectively, this body of evidence supports the notion that EPCs in their variable subtypes play an important role in the pathogenesis of diabetes. Thus, targeting the protective and reparative properties of these cells might constitute a therapeutic approach to preventing or limiting the vascular complications related to diabetes.

Despite the accepted and putative protective effects of metformin on the vascular endothelium, limited studies have investigated its effects on EPCs. Table **2** and Fig. (**1**) summarize the findings from the key studies. In clinical studies, metformin was found to improve the number and function of early EPCs, (proangiogenic cells and CFU-Hill’s colonies) in diabetic patients [79, 80]. Ahmed *et al.* proposed EPCs as markers of vascular repair and circulating endothelial cells as markers of vascular damage in T1D patients and have shown that metformin treatment corrects these levels [79]. This study further demonstrated that metformin enhances both the adhesion properties of proangiogenic EPCs and the homing ability to circulate EPCs, independently of glycemic control [79].

Combined therapy with metformin and the sulfonylurea, gliclazide, for 16 weeks was shown to have a more potent effect on the numbers and functions of EPCs in patients with T2D when compared to treatment with metformin alone [80]. This improvement was assumed to be linked to decreased oxidative stress as measured by reduced malonaldehyde levels and increased concentrations of plasma superoxide dismutase [80]. Combination therapy adding the dipeptidyl peptidase-4 (DPP-4) inhibitor, saxigliptin to concurrent metformin therapy for 12 weeks was found to increase CD31+ numbers (reflecting a mature circulating pool of endothelial cells) but not CD34+ in type II diabetes patients [81]. Additionally, the percentage of CD34+ EPCs expressing CXCR4 (SDF1α receptor) was increased, suggesting improved chemotaxis to SDF-1α and enhanced vasculogenic potential [81]. Therefore, the study suggested synergistic positive effects of saxigliptin and metformin on both mature and immature endothelial cells [81].

In animal models, metformin has also been found to improve EPC function and numbers [82-84]. Yu *et al.* showed that Sca-1+ Flk-1+ EPCs numbers were reduced in a type 1 diabetes mouse model and that treatment with metformin significantly increased cell numbers and improved angiogenesis and wound closure *in vivo* [82]. Additionally, treatment with metformin for 14 days increased the phosphorylation of AMPK and eNOS and enhanced NO generation in EPCs from animals with diabetes. This same group treated cultured bone marrow EPCs with high glucose media (33 mM) with or without metformin (2 mM) for 24 h and compared responses to control cells cultured in normal glucose media (5.5 mM). The study showed that metformin induced AMPK and eNOS phosphorylation and NO production and that these effects were inhibited by compound C (AMPK inhibitor), suggesting the involvement of the eNOS/AMPK pathway in the beneficial effects of metformin on EPCs. Similarly, Han *et al.* showed that treatment with metformin improved wound closure and capillary formation *in vivo* [83]. They also showed that bone marrow Sca-1+, Flk-1+ EPCs numbers were reduced in db/db diabetic and obese mice and treatment with metformin partially restored these numbers [83]. In addition, metformin improved tube formation ability, increased NO generation, and reduced intracellular oxygen concentration and thrombospondin-1 (TSP-1) in isolated EPCs.

Dallaglio **et al.*,* on the other hand, have shown that CD45- Sca1+ CD34+ CD31+ EPCs are increased in high-fat diet fed mice and that treatment with metformin reduced these numbers in white adipose tissue but had no effect on peripheral blood EPCs numbers in obese mice. This suggests that metformin might induce apoptosis in these progenitor cells or their differentiation to other cell types [84]. This contradiction in findings could be related to the differences in the isolated cells and/or different animal models.

*In vitro* experiments have shown that treatment of late EPCs with metformin (1mM) results in increased expression of CD31 and vWF, increased phosphorylation of eNOS and NO production and phosphorylation of AMPK, and decreased expression of mTOR and p70S6K phosphorylation [85]. Additionally, treatment of healthy late EPCs with metformin (10 mM) for 24 h reduced the expression of matrix metalloproteinase-2 and 9 and decreased EPC migration [86]. However, these effects were only observed using extremely high concentrations of metformin [85, 86]. Altogether, these findings indicate a therapeutic benefit of metformin on EPCs together with enhanced vascular repair mechanisms in diabetes. Further studies are, however, required to define the specific action(s) of clinically appropriate concentrations of metformin on each of the described phenotypes, which will ultimately aid in identifying specific targets to improve vascular repair [87].

## 4 METFORMIN AND AGEING

Data from studies of the effects of metformin on lifespan in the nematode, *Caenorhabditis elegans* (*C. elegans*), and also rodents provide insights on how age may account for some of the controversies in the literature concerning whether metformin has potential as a geroprotective agent. As previously mentioned, metformin protects endothelial cells against hyperglycaemia-induced senescence, suggesting a role for metformin as a senolytic agent [48]. Although there is a considerable database supporting the view that metformin extends lifespan in *C. elegans* [88, 89], it has also been reported that lifespan extension is not seen with older worms where, in fact, metformin has a negative effect and was linked to reduced mitochondrial numbers and function in the older worms [90]. Thus, age-dependent changes, particularly in cell metabolism, may reduce the protective effects of metformin and offset benefits related to its anti-hyperglycaemic and vascular protective actions when used in older subjects. Additional concerns relate to the high mM doses of metformin used in the studies to demonstrate life extension and also that the benefits of metformin have been linked to the effects on methionine metabolism in a specific *E. coli* strain, *E.coli*^OP50^, in the worm’s diet [88]. Comparable data have also been obtained when the effects of metformin are compared on the lifespan of young *versus* old rodents, with additional evidence showing that the effects of metformin are not equivalent to calorie restriction with the latter (but not metformin) associated with lifespan extension [91-93]. Glossmann and Lutz (2019) [94] and Mohammed *et al.* (2021) [57] have critically reviewed the evidence on whether metformin extends lifespan and extends health span in humans. Mohammed *et al.* defined healthspan as “*the period of life spent in good health and free of disabling diseases, or healthy lifespan”* [57]. The conclusion reached in the 2021 review by Mohammed *et al.* was:

*“The effects of metformin on healthspan are primarily indirect *via* its effects on cellular metabolism and result from its anti-hyperglycemic action, enhancing insulin sensitivity, reduction of oxidative stress and protective effects on the endothelium and vascular function”* [57].

Thus, whereas the use of metformin as an insulin-sensitizing and anti-hyperglycaemic drug has clear benefits in patients with T2D and reduces the impact of diabetes-relate premature aging of the vasculature while extending healthspan, it is less clear whether those benefits can be extended to non-T2D patients.

Collectively, these data also suggest that the use of metformin in older subjects, other than for those with T2D and when required to control hyperglycemia, should be carefully reviewed before initiating.

## 5 METFORMIN AND EXERCISE

Data from studies combining exercise with metformin treatment also raise concerns about whether metformin should be used in subjects without T2D and, again, indicates that the patient's age needs to be considered before initiating therapy. This is a particularly important consideration as exercise is considered to be the ‘Gold Standard’ for improving cardio-respiratory health with the view that ‘*Exercise can be considered as Medicine’* [95]. Thus, if indeed, the data from a number of studies show that the use of metformin reduces the beneficial effects of exercise on cardiovascular health, then this also argues for restricting the use of metformin to pathologies that require reducing insulin resistance and hyperglycaemia. There is substantive evidence that exercise positively affects many aspects of cardiovascular function [96]. Both exercise and metformin can improve glycemic control *via* the activation of AMPK, with AMPK described as an *“Exercise Mimetic”* [97]. Thus, it follows that metformin should also function as an exercise mimetic and combining metformin with exercise should provide at least additive effects, but this is not the case as data from a prospective, double-blinded, randomized, controlled study (RCT) indicates [98]. In this study, men and women with pre-diabetes followed an exercise protocol for 12 weeks with no drug as the placebo, *versus* metformin alone (2000 mg/day), *versus* a combination, or exercise plus placebo. The results indicated that although both metformin and exercise improved skeletal muscle insulin sensitivity by 55 and 90%, respectively the combination resulted in only a 30% enhancement [98]. The results were similar for effects on systolic BP and CRP that were reduced by 7 to 8% *versus* 20-25%, respectively; in addition, metformin blunted the exercise-induced increase in VO_2peak_ [99]. The authors speculate that the negative effect of metformin on exercise results from metformin lowering ROS levels, thus reducing the effects of ROS to activate AMPK, and suggesting that exercise, and not metformin, is the *“ideal drug”* [98]. A number of other studies have also provided data indicating that metformin attenuates the benefits of exercise [99-101]. In a randomized study with 53 older subjects (62 +/- 1 year), free of chronic diseases but with at least one risk factor for T2D, 27 were assigned to a 12-week aerobic exercise training (AET) plus metformin (2000, or 1500 mg/day for those experiencing GI side effects) group, *versus* 26 to the AET plus placebo group. In the group receiving metformin, exercise induced increases in whole-body insulin sensitivity were attenuated and exercise-induced increases in mitochondrial respiration were also reduced in patients with a family history or risk factors for T2D [99].

The double-blinded MASTERS (*Metformin to Augment Strength Training Effective Response in Seniors*) trial (NCT02308228) studied the effects of 2 weeks of metformin (1700 mg/day) *versus* placebo on a 14-week supervised progressive resistance exercise-training program (PRT) in 109 healthy men and women over the age of 65 with a mean age of 69.3 [101]. Compared to the placebo group, the 46 in the metformin group, despite an increase in AMPK signalling, showed a blunted exercise-induced hypertrophic response in thigh skeletal muscle mass in healthy men and significantly less total lean mass; essentially agreeing with an earlier study in subjects with pre-diabetes [101, 102]. The Look AHEAD (Action for Health in Diabetes) trial (NCT00017953) was designed to compare intensive lifestyle intervention *versus* diabetes support and education on outcomes of cardiovascular disease in T2D and based on data extracted from the study for 1982 individuals who used metformin together with either the lifestyle or education arms of the trial [100]. The results from the study showed that although the addition of metformin had a positive effect on reducing glycated hemoglobin (HbAIc), there was no additional benefit to cardiovascular fitness, and the use of metformin offsets the benefits of lifestyle intervention on weight loss [100]. Although there are differences in the duration of the treatment protocols of the various studies, collectively, the results indicate that the addition of metformin to exercise and lifestyle interventions provide no additional benefits to enhancing cardiovascular-respiratory health [99-102]. The results also complement those of the DPP previously discussed and demonstrate that the benefit of lifestyle intervention alone was greater than that resulting from metformin and the combination of the two interventions was less than lifestyle intervention alone [12, 13]. These results raise concerns about promoting the prescribing of metformin beyond its use as an anti-hyperglycemic drug.

## 6 VASCULAR BENEFITS OF METFORMIN *VERSUS* OTHER ANTI-DIABETIC DRUGS

A number of meta-analyses comparing metformin to the more recently introduced anti-diabetic drugs such as the GLP-1 receptor agonists (GLP-1 RAs), and dipeptidyl peptidase-4 (DPP-4) inhibitors and sodium-glucose co-transporter 2 (SGLT2) inhibitors do not consistently conclude that there is a positive effect of adding metformin. These observations raise the question as to whether metformin should remain the drug of choice to initiate therapy in patients with T2D or whether more aggressive multi-drug approaches should be initiated at an early stage [103, 104]. In addition, it was noted based on a meta-analysis of six trials of greater than 13,000 metformin-naïve T2D patients treated with either SGLT-2 inhibitors or GLP-1 RAs (3 trials with each class of drugs) that both groups of drugs reduced MACE [105]. The authors (Masson *et al.*, 2021) concluded: *“metformin would not be indispensable to obtain positive cardiovascular effects when new anti-diabetic drugs are administered.”* [105].

There are, however, potential added benefits to vascular function by combining metformin with other anti-diabetic drugs that should be further explored. Thus, Lunder *et al.* (2018) [106] compared the effects of empaglifozin (25 mg/day) to metformin alone (2000 mg/day) to empagliflozin plus metformin on arterial stiffness and brachial artery FMD in forty patients with T1D. The results showed that empagliflozin + metformin was superior to empagliflozin or metformin alone in improving FMD in patients with T1D, and empagliflozin alone and in combination with metformin improved arterial stiffness, whereas metformin alone had no effect on arterial stiffness [106].

It is also worthy of note that a meta-analysis of patients treated with SGLT-2 inhibitors indicates the benefit of this class of drugs in reducing hospitalization due to heart failure [107], which was also a conclusion of the analysis by Masson *et al.*, (2021) [105]. Of related interest, Salvatore *et al.*, (2021) [108] argue for the need for appropriate CV outcome trials to determine if metformin treatment of subjects with T2D helps reduce the risk of the development of heart failure. Schernthaner *et al.*, (2022) have reviewed the current status of metformin in the treatment of patients with heart failure [109]. Of particular interest is the on-going DANEHEART trial (NCT03514108), which is a 4-year study involving 1500 patients with T2D and heart failure comparing metformin with hydralazine/isosorbide dinitrate, or placebo [110]. The results of the DANEHEART study are expected in 2023.

A direct comparison of metformin with the newer anti-diabetic drugs is complicated by the so-called “legacy effect” noted in the UKPDS, wherein the CV benefits of metformin were observed on follow-up after > 10 years, thus making direct comparisons with data from trials of shorter durations of less than 3 to 5 years questionable [8, 108]. Furthermore, and as discussed, information is now available showing positive clinical trial data with the SGLT-2 inhibitors (gliflozins) and GLP-1 RAs and their CV protective benefits in patients with T2D, and like metformin, their use in non-diabetic populations is being considered. However, as evident from the UK’s Clinical Practice Research Datalink, metformin was the drug of choice for more than 70% of patients with T2D, regardless of whether CVD was a comorbidity [111]. Another factor to consider is that for the UKPDS trial, the first-generation sulfonylurea, chlorpropamide, was one of the two sulfonylureas prescribed and linked to its comparative long plasma half-life is associated with a greater risk of hypoglycaemia; but today, second- and third generation agents, glimepiride and glipizide, are more frequently used. These are more potent and considered safer. For instance, see the Cochrane systematic review and meta-analysis data that shows that third-generation drugs reduce non-fatal macrovascular outcomes [112]. Finally, the UKPDS 49 report [113] made the conclusion that to achieve HbAIc below 7.8 mmol/L, 50% of patients within 3 years of diagnosis required more than one anti-diabetic drug. This requirement is also a consideration when discussing the potential benefits of prescribing metformin to subjects with CVD but without diabetes and considering the duration of the treatment as well as the other drugs that the patient has been prescribed.

### 6.1 Metformin and the Kidney

Considerable interest has developed in the use of metformin in the treatment of kidney disease, including that unrelated to diabetes (for example, polycystic renal disease, nephritis and acute kidney injury). Evidence from both clinical trials and experimental animal models of kidney disease have reported beneficial actions of metformin. While concern and debate have been raised as to its use in subjects with severe renal impairment due to the possibility of lactic acidosis (for example, see Broe and Jouret, 2020 [114] and Hanna *et al.*, 2020 [115]), metformin use has been noted to be safe by the US FDA in those with mild to moderate renal impairment (eGFR > 30ml/min). Further, there is growing recognition that while there may be a low risk of lactic acidosis with metformin treatment, this risk can be even further reduced by adjusting the dosage, thus adding to the potential for using metformin in patients with renal disease [116-118].

In a retrospective study of patients with type 2 diabetes and an initial eGFR > 60 ml/minute/1.73 m^2^, metformin treatment provided a lower risk for the decline of kidney function or death compared to those treated with sulfonylurea [119]. Importantly, the beneficial effects of metformin occurred independently of changes in body mass index, blood pressure and glycemic control, as evidenced by glycated hemoglobin levels [119]. In a meta-analysis of 17 observational studies, Crowley *et al.* (2017) [120] reported that in subjects with type 2 diabetes and chronic kidney disease (CKD; defined as eGFR < 60 mL/min/1.73 m^2^), congestive heart failure or chronic liver disease, metformin treatment was associated with a reduction in all-cause mortality compared to diabetic subjects not receiving metformin. Further, metformin has been suggested to exert renoprotective effects in CKD patients undergoing kidney transplants, as shown by increased allograft and patient survival [121].

Studies in animal models of acute kidney disease and CKD similarly support a renoprotective role for metformin. For example, administration of metformin has been shown to decrease renal fibrosis and proteinuria in kidney disease induced by cyclosporin A [122], 0.25% adenine diet [123], and sub-total nephrectomy [124]. Similarly, metformin is renoprotective in rats rendered insulin resistant by high fructose consumption [125].

**Fig. (2):**
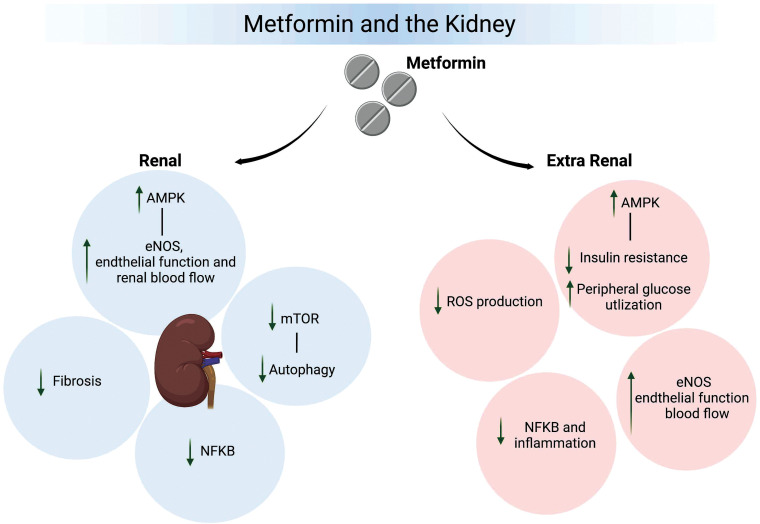
Does metformin protect kidney function? Concerns over hepatotoxicity and lactic acidosis resulted in the almost universal withdrawal in 1978 of the biguanides phenformin and busformin from clinical use. Metformin inherited these concerns, although the incidence of lactic acidosis associated with the use of metformin is very low, with the risk greatly reduced by avoiding use in patients with severe chronic kidney disease (CKD) where eGFR is <30 mL/min/1.73 m^2^. As depicted in the figure, an accumulation of evidence from both pre-clinical and clinical studies supports the view that metformin has both indirect and direct renoprotective actions. Extra-kidney protective effects can be linked, at least in part, to the activation of AMPK, reduction of insulin resistance, enhanced peripheral glucose utilization, and protection of vascular function. Evidence, primarily from studies with rodent models of diabetes, also supports direct renoprotective effects of metformin that involve the activation of AMPK. The downstream effects of AMPK protect multiple cell types in the kidney *via* enhanced endothelial/vascular function, reduced NFκB activation, inhibition of mammalian target for rapamycin (mTOR) and autophagy, and reduced inflammation and fibrosis. This figure was created with BioRender.

As in other tissues, the effects of metformin are suggested to involve both AMPK - dependent and - independent actions (Fig. **2**). Underlying mechanisms have similarly been suggested to involve a number of likely interrelated events, including anti-oxidant and - inflammatory effects, attenuation of pathways leading to renal fibrosis, normalization of cellular metabolism (including impaired insulin signaling), and attenuation of hypoxia and modulation of renal/glomerular hemodynamics. Importantly, while a number of these actions may occur directly at the level of renal cells, the kidney will likely be impacted by extra-renal factors, including systemic inflammation, ROS production and altered hemodynamics (Fig. **2**).

A complete understanding of the direct actions of metformin on the kidney is complicated by possible effects on multiple cell types, including vascular cells, tubular epithelial cells, podocytes and mesangial cells. *in vitro* studies of specific cell types aimed at delineating mechanisms necessarily obscure interactions between these cells. Further, *in vitro* studies of cultured renal cells are often conducted in the presence of very high metformin concentrations (mM), although an argument can be made that existing renal impairment could result in high local drug levels.

Additional information on the specific effects of metformin in the kidney and in the presence of varying levels of kidney disease can be found in recent articles, including Corremans *et al.*, 2018 [116]; Salvatore *et al.*, 2019 [118]; Pan *et al.*, 2020 [126]; and Song *et al.*, 2021 [127]. Nevertheless, the available clinical and experimental data support a need for further focused clinical and experimental studies, particularly given the current interest in the impact of novel anti-diabetes agents (*e.g.* SGLT2 inhibitors) on both heart failure and CKD.

## 7 METFORMIN AND COVID-19

It became evident early in the COVID-19 pandemic that patients with underlying co-morbidities were at higher risk of hospitalization. Specifically, the risk was particularly high in diabetes, with diabetes patients being approximately twice as likely to die from COVID-19 [128-131]. The risk is further increased in those patients with higher BMIs, thus emphasizing the contribution of metabolic dysfunction to the severity of COVID-19 [132]. As detailed by Drucker (2021) [133], such patients will have a heightened level of baseline inflammation with resultant thromboinflammation, endothelial dysfunction, and hypercoagulation. These clinical signs suggest that a drug like metformin with a proven history as an effective and safe anti-hyperglycaemic drug and endothelial protective should prove beneficial.

Hyperglycaemia, regardless of whether associated with diabetes, has been shown to be associated with adverse outcomes [134, 135], and lower mortality is associated in patients with better glycemic control [136]. The importance of monitoring and controlling blood glucose levels in COVID-19 was emphasized in 2020 by an editorial by Ceriellio [137]: *“Hyperglycemia and the worse prognosis of COVID-19. Why a fast blood glucose control should be mandatory.”* The link between hyperglycaemia and the severity of COVID-19 has also been emphasized by data indicating that approximately 50% of 3854 patients with COVID-19 had glucose levels greater than 9.4 mmol/L, and over 90% of intubated COVID-19 patients were hyperglycaemic with elevated C-peptide levels [138]. It was also reported that adiponectin levels were lower, thus inferring adipose dysfunction [138]. An analysis in South Korea of over 4000 COVID-19 patients assessed the contribution of the patient's metabolic health on outcomes [139, 140]. COVID-19 patients were categorised in metabolically healthy normal weight *versus* metabolically unhealthy normal weight *versus* metabolically healthy obese *versus* metabolically unhealthy obese, where unhealthy was determined based on fasting glucose, BP, serum triglycerides and lipids. Analysis by Kim *et al.* revealed that metabolic health is more important than obesity as a determinant of the health outcomes of COVID-19 patients [139]; see also editorial Sanoudou *et al.*, 2022 [140].

Of interest are the results from retrospective studies and systematic reviews that COVID-19 patients treated with metformin have reduced mortality [141-145]. The anti-inflammatory effects of metformin have been attributed to the suppression of IL6 and TNFα and previously described [141, 146, 147]. Cameron *et al.* (2016) presented data from both human studies and from a mouse hepatocyte cell culture protocol [147]. The data from hepatocytes indicated that a 3 h exposure to a supra-pharmacological concentration of 2 mM metformin reduced TNFα activation of NF-κB activation. This high concentration of metformin, which may well inhibit mitochondrial complex 1, is not found in metformin-treated individuals. For the human studies, data were derived from a clinical trial of non-diabetic heart failure (NCT00473876 Metformin in Insulin Resistant Left Ventricular Dysfunction), and analysis based on that a reduction in the neutrophil to lymphocyte ratio (NLR) is a measure of anti-inflammatory action of metformin. Based on changes in the NLR Cameron *et al.*, concluded that the effects of metformin were independent of modulating glucose homeostasis [147]. However, it is unclear whether there are particular benefits associated with metformin *versus* other anti-hyperglycemic drugs or whether the benefits are directly associated with the anti-hyperglycaemic efficacy of the drug. In the study described by Cameron *et al.*, the NLRs for patients receiving either metformin *versus* a sulfonylurea were below 3, 1.94 to 2.56, respectively, inferring that sulfonylureas may also provide protection against inflammation. Furthermore, as pointed out above, the mouse hepatocyte data were based on the use of 2 mM metformin [147, 148]. Based on an interactome analysis of a large number of drugs for potential repurposing to treat COVID-19, metformin in the concentration range of 10 nM to 100 μM was shown to have no effect on viral growth in Vero E6 cells transfected with SARS-CoV-2 [149]. In the absence of any compelling evidence that metformin possesses any significant direct anti-viral activity, it has been argued that its effectiveness is mediated through activation of AMPK and modulation of many of the downstream targets of AMPK, thereby reducing the severity of COVID-19. Such targets include inhibition of the mammalian target for rapamycin (mTOR) and phosphorylation of the host cell target for the virus, ACE2 (angiotensin converting enzyme 2), thereby reducing the ability of the virus to infect the host, as was also reported with MERS [150-152]. It is also likely that the ability of metformin to preserve endothelial function in an inflammatory setting can reduce the thromboinflammatory effect of infection with SARS-CoV-2. Pulmonary microthrombi are known to play a role in the pathophysiology of COVID-19 and result from the virus invading the endothelium *via* ACE2, which is richly expressed in endothelial cells [153]. Endotheliitis resulting from the direct infection of endothelial cells with SARS-CoV-2 has been demonstrated with evidence of resultant endothelial cell death [154]. These putative benefits of metformin in a patient with COVID-19 are summarized in Fig. (**3**).

Importantly, essentially all classes of drugs that are used to treat hyperglycaemia and lower blood glucose levels will also reduce pro-inflammatory markers such as CRP, IL-6 and ferritin [155, 156]. Furthermore, data from Wuhan indicated that COVID-19 patients who maintained good glycemic control fared better [136]. In addition, a study from Spain reported no association between anti-diabetic drug use and adverse outcomes or mortality in COVID-19 patients [157]. The French Coronavirus-SARS-CoV-2 and Diabetes Outcomes (CORONADO) study found that younger COVID-19 patients fared better since they had fewer severe co- morbidities and were treated with metformin [144, 158]. Finally, the conclusion based on an observational cohort study utilizing the UK’s National Diabetes Audit that assessed the risk of different glucose lowering drugs in 2.8 million T2D patients was that although a majority were receiving metformin, there was no clear indication to change the anti-diabetes agent prescribed to the patient with COVID-19 [159]. A breakdown of the drugs prescribed to the patients reveals that 63.1% received metformin; 19.7% a sulfonylurea; 9.3% an SGLT2 inhibitor; DPP-4 inhibitors; 16.8%, and 12.3% insulin, but only 2.1% with a thiazolidinedione and 3.9% a GLP-1 agonist [159]. Of particular interest is that those prescribed metformin, SGLT2 inhibitors, and sulfonylureas had a statistically lower mortality risk than those prescribed insulin or a DPP-4 inhibitor; these drugs also have diverse non-overlapping cellular modes of action to lower hyperglycaemia. It is also well established that metformin is the first drug prescribed to the majority of newly diagnosed patients with T2D, whereas insulin is given to patients with more advanced T2D [159]. Collectively, these data indicate that the most likely basis of the effectiveness of metformin, SGLT-2 inhibitors and sulfonylureas in reducing the risk of severe COVID-19 is linked to their anti-hyperglycemic actions rather than to unrelated pleiotropic actions. This conclusion is supported by editorials [109, 160]. There are, however, patient-specific and physician-specific as well as cost reasons why one particular anti-diabetic drug may be the less preferred choice. For metformin, this could be due to persistent GI problems or the, albeit low, risk of lactic acidosis, particularly in the elderly if the patient has impaired renal and liver function. For SGLT2 inhibitors there are concerns over dehydration and euglycemic ketoacidosis, and anorexia with GLP-1 RAs [161].

Although appropriately designed RCTs would help to resolve the question as to whether there is a particular benefit provided by metformin *versus* other anti-hyperglycaemic drugs and it is likely that the proven endothelial-protective actions of metformin, albeit at least in part related to its anti-hyperglycaemic actions, contribute to its beneficial effects in COVID-19 patients.

## CONCLUSION

Metformin has been in clinical use for the treatment of T2D for over 60 years, and since 1998, with the release of the data from the UKPDS, it has been the first-choice drug for patients. This long history of use has also provided a substantial database that not only credits the drug as a safe and effective for the treatment of T2D but also has additional benefits in terms of CV protection. *Via* its action as an insulin-sensitizer, metformin has also proven useful to treat PCOS, and data, albeit controversial, indicates benefits in reducing the risk of some cancers; the latter may also depend on its actions as an insulin sensitizer and enhancing glucose utilization. In addition to its action on glucose homeostasis, the proven endothelial-vascular protective effects of metformin may contribute to the benefits seen in the treatment of patients with COVID-19, as well as claims that metformin offsets the advance of age-related diseases. However, given the CV benefits of metformin in the treatment of patients with T2D, surprisingly, its usefulness in TID is very limited. Furthermore, in the absence of data from appropriately designed and powered RCTs, there are also concerns about over-extending the use of metformin beyond its role as an insulin-sensitizer and anti-hyperglycaemic drug. In part, these concerns are based on lingering concerns over the risk of lactic acidosis, albeit over-emphasized, and also concerns over extrapolation from pre-clinical data that has been generated with high concentrations of metformin. Further, the data from investigations such as the MASTERS study [101] indicated that metformin reduced the benefits of exercise in adults over the age of 65. In addition, and as emphasized in a report from the Western France Poison Control Centre in Nantes, the risk of both accidental and intentional poisoning with metformin should not be under-estimated [162] and may increase if the drug becomes more widely used. We conclude that metformin has proven CV benefits in patients with T2D that might extend to protect against vascular disease in the absence of a diagnosis of diabetes; however, caution is appropriate before expanding the use of metformin as a prophylactic to otherwise healthy patients.

## Figures and Tables

**Fig. (1):**
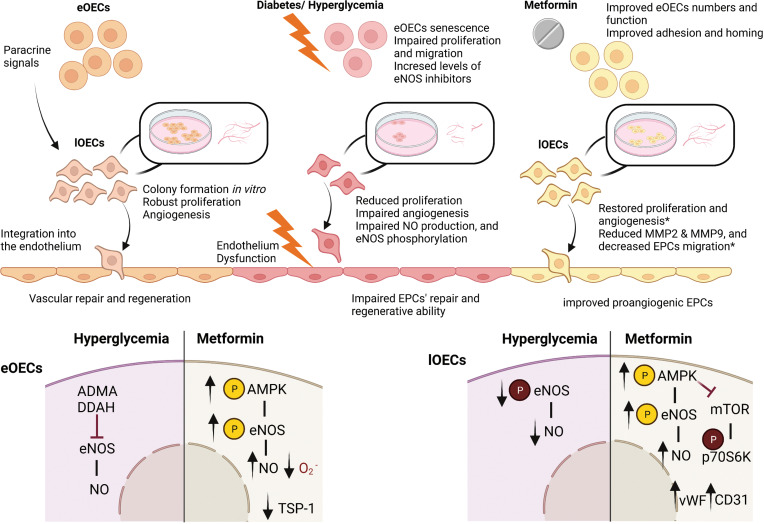
The protective effect of metformin on EPCs in diabetes. Early (eOECs) and late (lOECs) EPCs play a synergistic role in vascular repair and regeneration. eOECs are involved in vascular repair through the paracrine regulation of neovascularization, while lOECs respond to these paracrine signals and incorporate them into the site of injury. Diabetes and hyperglyacemia affect the numbers and functions of EPCs. Treatment with metformin restores some of these functions. *supra-pharmacological concentrations. Figure created with BioRender.

**Fig. (3):**
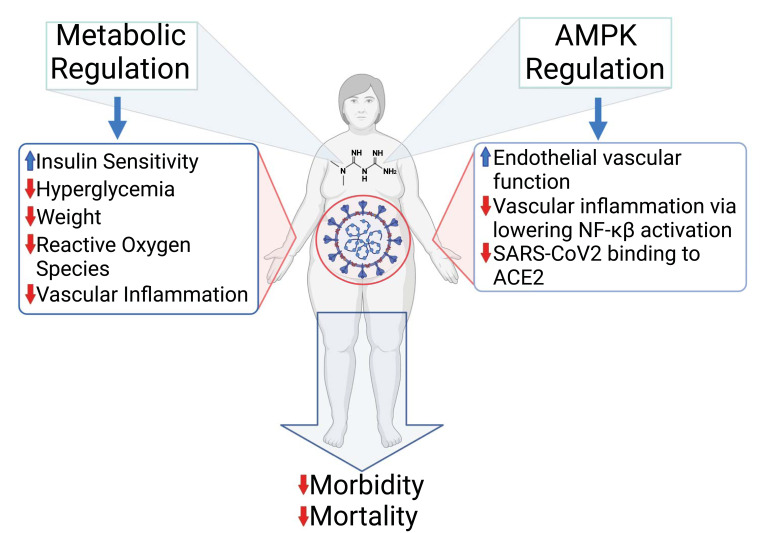
The clinical data indicate that patients with diabetes and who are also obese are at higher risk of suffering serious complications and reduced survival if infected with the SARS-CoV-2 virus. Use of metformin has been shown to reduce both morbidity and mortality associated with COVID-19, although these benefits seem to be variably shared with other anti-diabetic drugs that have diverse mechanisms of action and cellular targets. As depicted under ‘Metabolic Regulation’ (left side of figure), metformin by virtue of its insulin sensitizing action and enhanced cellular uptake of glucose, reduces the impact of hyperglycaemia and resultant oxidative stress that would otherwise enhance vascular inflammation and promote endothelial dysfunction. As depicted on the right side of the figure, metformin, *via* the activation of AMPK, potentially may enhance eNOS activity, protect and enhance endothelial function, reduce activation of Nf-κB and lower endothelial-vascular inflammation. In addition, AMPK has been reported to phosphorylate ACE2, thereby hindering the ability of the SARS-CoV-2 virus to bind to its receptor and reduce the cellular entry of the virus and subsequent infection. Collectively, these actions of metformin reduce the impact of COVID-19 on morbidity and mortality. This figure was created with BioRender.com.

**Table 1:** A comparison between early and late EPCs.

-	**Early EPCs**	**Late EPCs**
Other Terminology	Early outgrowth endothelial cells (eOECs), Endothelial cell colony-forming units (CFU-ECs), myeloid angiogenic cells (MAC), also called circulating angiogenic cells (CAC)	Late outgrowth endothelial cells (lOECs), blood outgrowth endothelial cells (BOECs), Endothelial colony forming cells (ECFCs)
Emergence in Culture	Cultured on fibronectin coated plates, emerge in 5-7 days	Cultured on collagen coated plates, emerge in 7-21 days
Morphology	Spindled Shape	Cobblestone Morphology
Surface Markers	CD31, CD45, and CD14, and lack expression of CD133	CD34, CD31, and CD133 and lack the expression of the hematopoietic markers CD45, CD14, and CD115
Origin	Hematopoietic	Unclear, suggested to originate from tissue vascular niches
Angiogenic Potential	Capable	Highly Capable
Incorporation into vessels	Incapable	Highly Capable
Migration	Capable	Capable
Cytokine Release	High	Low
Phagocytic Function	Capable	Incapable
Differentiation to mature endothelial cells	Incapable	Capable
Involvement in vascular repair	Paracrine	Active participation by incorporation into vessels to promote vascular repair

**Note:** *[67, 68, 78].

**Table 2:** Summary of studies investigating the effects of metformin on EPCs.

-	**Study**	**EPC Subtype**	**Main Findings**
Early EPCs and FACS Isolated EPCs	Ahmed *et al.,* 2016 [79]	Cells were isolated from the peripheral blood of T1D patients. EPCs quantification by FACS (circulating EPCs were defined as CD45^dim^ CD34+VEGFR-2+ cells and cECs as CD45^dim^, CD133−, CD34+, and CD144+ cells). Also, isolation of proangiogenic cells and CFU-Hill’s (early EPCs).	Metformin (1000 mg/bid) treatment of T1D patients for 8 weeks improved circulating EPCs, proangiogenic cells, CFU-Hill’s colony numbers and function.
	Chen *et al.,* 2010 [80]	Early EPCs (CFU) isolated by selective plating, and cells isolated by FACS (CD45^low^/CD34+/VEGFR2+).	Metformin alone (500-2500 mg/day in patients), and metformin + gliclazide (a sulfonylurea) combination treatment improved the number and function of EPCs in patients with T2D over 16 weeks (short term hypoglycemic treatment).
	Dore *et al.,* 2018 [81]	CD34+ cells were isolated by CD34+ microbead antibody protocol and counted by FACS from 42 patients with T2D diagnosed within 10 years. Also, isolation of CFU-Hill’s colonies.	Saxigliptin for 12 weeks added to concurrent metformin therapy increased CD31+ numbers (reflecting a mature circulating pool of endothelial cells) but not CD34+. Double positive cells for CD34 and CXCR4 were increased with dual treatments.
	Dallaglio *et al.,* 2014 [84]	FACS enumeration of CD45- Sca1+ CD34+ CD31+ EPCs in peripheral blood and visceral white adipose tissue of obese mice receiving a high-fat diet (0.5mg metformin/ml orally).	EPCs were increased in high-fat diet fed mice, and treatments with metformin reduced these numbers in white adipose tissue but had no effect on peripheral blood EPCs only in obese mice.
	Yu *et al.,* 2016* [82]	Bone marrow Sca-1+ Flk-1+ EPCs were isolated from the blood of type 1 diabetes mice and treated with metformin (250 mg/kg/d, intragastric) by FACS. Also, selective plating of EPCs on vitronectin coated plates.	Metformin significantly increased the number of Sca-1+ Flk-1+ EPCs in diabetic mice improved angiogenesis and wound closure *in vivo*. Metformin increased the expression of phosphorylated-AMPK and eNOS and the generation of NO in diabetic EPCs. *In vitro* experiments: Cultured bone marrow EPCs were treated with high glucose media (33 mM) with or without metformin (2 mM) for 24 h and compared to controls cultured in normal glucose media (5.5 mM). Metformin (2 mM) induced AMPK and eNOS phosphorylation and the generation of NO.
	Han *et al.,* 2016 [83]	Genetically hyperglycaemic db/db mice were treated with metformin (250 mg/kg/day intragastric) for 2 weeks. FACS enumerates Sca-1+ and Flk-1+ cells. Also, selective plating on vitronectin coated plates.	Treatment with metformin improved wound closure and capillary formation *in vivo*. EPCs numbers were reduced in db/db mice, and treatments with metformin partially restored these numbers. In mice treated with metformin, tube formation ability of EPCs was induced in addition to an increase in NO generation and a reduction in intracellular oxygen concentration and TSP-1.
Late EPCs	Li *et al.*, 2015* [85]	Bone marrow EPCs isolated from healthy rats by selective plating. Colonies appeared at day 7 and reached confluency at day 14.	Metformin (1mM) regulates EPCs differentiation to endothelial cells through autophagy and AMPK-eNOS-NO pathways. Treatment with metformin resulted in an increased expression of CD31 and vWF, increased phosphorylation of AMPK, and decreased expression of mTOR and p70S6K phosphorylation. Metformin concentrations between 2-10 mM inhibited EPC proliferation following 48 h of treatment, while concentrations between 0.5-1mM had no effect on proliferation.
	Li *et al., *2017* [86]	Late EPCs isolated by selective plating on collagen coated plates.	Metformin treatment (10 mM) of healthy EPCs reduced the expression of matrix metalloproteinase-2 and 9, and decreased EPCs migration.

**Note:** *Attention is drawn to the high concentrations of metformin that were used in some of the listed *in vitro* studies. Peak plasma levels of metformin, when used clinically in humans, are unlikely to be higher than 20 μM, although higher levels of 50 μM might be initially achieved in the portal circulation immediately after absorption [87]. With a half-life of between 4-5 h and administered either once daily or maximally thrice, studies on the effects of metformin should ideally be performed with concentrations no higher than 20 μM.

## LIST OF ABBREVIATIONS

ACE2: Angiotensin Converting Enzyme 2
AMPK: AMP Activated Protein Kinase
BP: Blood Pressure
COVID-19: Coronavirus Disease 19
CRP: C Reactive Protein
CV: Cardiovascular
CVD: Cardiovascular Disease
DPP-4: Dipeptidyl Peptidase-4
EDV: Endothelial-dependent Vasodilation
eNOS: endothelial Nitric Oxide Synthase
eOECs: Early Outgrowth Endothelial Cells
EPC: Endothelial Progenitor Cells
FACS: Fluorescence-activated Cell Sorter
FMD: Flow Mediated Dilatation
GLP-1: Glucagon-like Peptide-1
GLP-1 RA: Glucagon-like Peptide-1 Receptor Agonist
HbA1c: Glycated Haemoglobin
ICAM-1: Intercellular Adhesion Molecule-1
LKB1: Liver Kinase B1
lOECs: Late Outgrowth Endothelial Cells
MACE: Major Adverse Cardiac Event
MERS: Middle East Respiratory Syndrome
mTOR: Mammalian Target for Rapamycin
NO: Nitric Oxide
PCOS: Polycystic Ovary Syndrome
ROS: Reactive Oxygen Species
RCT: Randomized Controlled Trial
SARS-CoV-2: Severe Acute Respiratory Syndrome Coronavirus 2
SGLT-2: Sodium Glucose Co-Transporter-2
T1D: Type 1 Diabetes
T2D: Type 2 Diabetes
TSP-1: Thrombospondin-1
UKPDS: United Kingdom Prospective Diabetes Study
VCAM-1: Vascular Cell Adhesion Molecule-1
VEGF: Vascular Endothelial Growth Factor
vWF: Von Willebrand Factor

## References

[1] Sterne J. (1957). Du nouveau dans les antidiabetiques. La NN dimethylamine guanyl guanide (NNDG).. Maroc Med..

[2] Glossmann H.H., Lutz O.M.D. (2019). Pharmacology of metformin - An update.. Eur. J. Pharmacol..

[3] Zhou G., Myers R., Li Y., Chen Y., Shen X., Fenyk-Melody J., Wu M., Ventre J., Doebber T., Fujii N., Musi N., Hirshman M.F., Goodyear L.J., Moller D.E. (2001). Role of AMP-activated protein kinase in mechanism of metformin action.. J. Clin. Invest..

[4] Muise E.S., Guan H-P., Liu J., Nawrocki A.R., Yang X., Wang C., Rodríguez C.G., Zhou D., Gorski J.N., Kurtz M.M., Feng D., Leavitt K.J., Wei L., Wilkening R.R., Apgar J.M., Xu S., Lu K., Feng W., Li Y., He H., Previs S.F., Shen X., van Heek M., Souza S.C., Rosenbach M.J., Biftu T., Erion M.D., Kelley D.E., Kemp D.M., Myers R.W., Sebhat I.K. (2019). Pharmacological AMPK activation induces transcriptional responses congruent to exercise in skeletal and cardiac muscle, adipose tissues and liver.. PLoS One.

[5] UK Prospective Diabetes Study (UKPDS) Group (1998). Intensive blood-glucose control with sulphonylureas or insulin compared with conventional treatment and risk of complications in patients with type 2 diabetes (UKPDS 33).. Lancet.

[6] UK Prospective Diabetes Study (UKPDS) Group (1998). Effect of intensive blood-glucose control with metformin on complications in overweight patients with type 2 diabetes (UKPDS 34).. Lancet.

[7] Stratton I.M., Adler A.I., Neil H.A., Matthews D.R., Manley S.E., Cull C.A., Hadden D., Turner R.C., Holman R.R. (2000). Association of glycaemia with macrovascular and microvascular complications of type 2 diabetes (UKPDS 35): Prospective observational study.. BMJ.

[8] Holman R.R., Paul S.K., Bethel M.A., Matthews D.R., Neil H.A. (2008). 10-year follow-up of intensive glucose control in type 2 diabetes.. N. Engl. J. Med..

[9] Johnson J.A., Simpson S.H., Toth E.L., Majumdar S.R. (2005). Reduced cardiovascular morbidity and mortality associated with metformin use in subjects with type 2 diabetes.. Diabet. Med..

[10] Jong C.B., Chen K.Y., Hsieh M.Y., Su F.Y., Wu C.C., Voon W.C., Hsieh I.C., Shyu K.G., Chong J.T., Lin W.S., Hsu C.N., Ueng K.C., Lai C.L. (2019). Metformin was associated with lower all-cause mortality in type 2 diabetes with acute coronary syndrome: A Nationwide registry with propensity score-matched analysis.. Int. J. Cardiol..

[11] Bergmark B.A., Bhatt D.L., McGuire D.K., Cahn A., Mosenzon O., Steg P.G., Im K., Kanevsky E., Gurmu Y., Raz I., Braunwald E., Scirica B.M., SAVOR-TIMI 53 Steering Committee and Investigators (2019). Metformin use and clinical outcomes among patients with diabetes mellitus with or without heart failure or kidney dysfunction: Observations from the SAVOR-TIMI 53 Trial.. Circulation.

[12] Knowler W.C., Barrett-Connor E., Fowler S.E., Hamman R.F., Lachin J.M., Walker E.A., Nathan D.M., Diabetes Prevention Program Research Group (2002). Reduction in the incidence of type 2 diabetes with lifestyle intervention or metformin.. N. Engl. J. Med..

[13] Diabetes Prevention Program Research Group (2012). Long-term safety, tolerability, and weight loss associated with metformin in the Diabetes Prevention Program Outcomes Study.. Diabetes Care.

[14] Han Y., Xie H., Liu Y., Gao P., Yang X., Shen Z. (2019). Effect of metformin on all-cause and cardiovascular mortality in patients with coronary artery diseases: A systematic review and an updated meta-analysis.. Cardiovasc. Diabetol..

[15] Griffin S.J., Leaver J.K., Irving G.J. (2017). Impact of metformin on cardiovascular disease: A meta-analysis of randomised trials among people with type 2 diabetes.. Diabetologia.

[16] Boussageon R., Supper I., Bejan-Angoulvant T., Kellou N., Cucherat M., Boissel J-P., Kassai B., Moreau A., Gueyffier F., Cornu C. (2012). Reappraisal of metformin efficacy in the treatment of type 2 diabetes: A meta-analysis of randomised controlled trials.. PLoS Med..

[17] Boussageon R., Gueyffier F., Cornu C. (2016). Metformin as firstline treatment for type 2 diabetes: Are we sure?. BMJ.

[18] Furchgott R.F., Zawadzki J.V. (1980). The obligatory role of endothelial cells in the relaxation of arterial smooth muscle by acetylcholine.. Nature.

[19] Triggle C.R., Ding H., Marei I., Anderson T.J., Hollenberg M.D. (2020). Why the endothelium? The endothelium as a target to reduce diabetes-associated vascular disease.. Can. J. Physiol. Pharmacol..

[20] Verma S., Anderson T.J., Fundamentals of Endothelial Function for the Clinical Cardiologist (2002). Fundamentals of endothelial function for the clinical cardiologist.. Circulation.

[21] Vita J.A., Keaney J.F. (2002). Endothelial function: A barometer for cardiovascular risk?. Circulation.

[22] Anderson T.J., Uehata A., Gerhard M.D., Meredith I.T., Knab S., Delagrange D., Lieberman E.H., Ganz P., Creager M.A., Yeung A.C. (1995). Close relation of endothelial function in the human coronary and peripheral circulations.. J. Am. Coll. Cardiol..

[23] Little P.J., Askew C.D., Xu S., Kamato D. (2021). Endothelial dysfunction and cardiovascular disease: History and analysis of the clinical utility of the relationship.. Biomedicines.

[24] Nafisa A., Gray S.G., Cao Y., Wang T., Xu S., Wattoo F.H., Barras M., Cohen N., Kamato D., Little P.J. (2018). Endothelial function and dysfunction: Impact of metformin.. Pharmacol. Ther..

[25] Ding H., Ye K., Triggle C.R. (2019). Impact of currently used anti-diabetic drugs on myoendothelial communication.. Curr. Opin. Pharmacol..

[26] Ding Y., Zhou Y., Ling P., Feng X., Luo S., Zheng X., Little P.J., Xu S., Weng J. (2021). Metformin in cardiovascular diabetology: A focused review of its impact on endothelial function.. Theranostics.

[27] Salvatore T., Pafundi P.C., Galiero R., Rinaldi L., Caturano A., Vetrano E., Aprea C., Albanese G., Di Martino A., Ricozzi C., Imbriani S., Sasso F.C. (2020). Can metformin exert as an active drug on endothelial dysfunction in diabetic subjects?. Biomedicines.

[28] Mather K.J., Verma S., Anderson T.J. (2001). Improved endothelial function with metformin in type 2 diabetes mellitus.. J. Am. Coll. Cardiol..

[29] Vitale C., Mercuro G., Cornoldi A., Fini M., Volterrani M., Rosano G.M. (2005). Metformin improves endothelial function in patients with metabolic syndrome.. J. Intern. Med..

[30] de Jager J., Kooy A., Lehert P., Wulffelé M.G., van der Kolk J., Bets D., Verburg J., Donker A.J., Stehouwer C.D. (2010). Long term treatment with metformin in patients with type 2 diabetes and risk of vitamin B-12 deficiency: Randomised placebo controlled trial.. BMJ.

[31] de Jager J., Kooy A., Schalkwijk C., van der Kolk J., Lehert P., Bets D., Wulffelé M.G., Donker A.J., Stehouwer C.D. (2014). Long-term effects of metformin on endothelial function in type 2 diabetes: A randomized controlled trial.. J. Intern. Med..

[32] Sirtori C.R., Franceschini G., Gianfranceschi G., Sirtori M., Montanari G., Bosisio E., Mantero E., Bondioli A. (1984). Metformin improves peripheral vascular flow in nonhyperlipidemic patients with arterial disease.. J. Cardiovasc. Pharmacol..

[33] Sirtori C.R., Franceschini G., Galli-Kienle M., Cighetti G., Galli G., Bondioli A., Conti F. (1978). Disposition of metformin (N,N-dimethylbiguanide) in man.. Clin. Pharmacol. Ther..

[34] de Aguiar L.G., Bahia L.R., Villela N., Laflor C., Sicuro F., Wiernsperger N., Bottino D., Bouskela E. (2006). Metformin improves endothelial vascular reactivity in first-degree relatives of type 2 diabetic patients with metabolic syndrome and normal glucose tolerance.. Diabetes Care.

[35] Pitocco D., Zaccardi F., Tarzia P., Milo M., Scavone G., Rizzo P., Pagliaccia F., Nerla R., Di Franco A., Manto A., Rocca B., Lanza G.A., Crea F., Ghirlanda G. (2013). Metformin improves endothelial function in type 1 diabetic subjects: A pilot, placebo-controlled randomized study.. Diabetes Obes. Metab..

[36] Rena G., Lang C.C. (2018). Repurposing metformin for cardiovascular disease.. Circulation.

[37] Jadhav S., Ferrell W., Greer I.A., Petrie J.R., Cobbe S.M., Sattar N. (2006). Effects of metformin on microvascular function and exercise tolerance in women with angina and normal coronary arteries: A randomized, double-blind, placebo-controlled study.. J. Am. Coll. Cardiol..

[38] Preiss D., Lloyd S.M., Ford I., McMurray J.J., Holman R.R., Welsh P., Fisher M., Packard C.J., Sattar N. (2014). Metformin for non-diabetic patients with coronary heart disease (the CAMERA study): A randomised controlled trial.. Lancet Diabetes Endocrinol..

[39] Luo F., Das A., Chen J., Wu P., Li X., Fang Z. (2019). Metformin in patients with and without diabetes: A paradigm shift in cardiovascular disease management.. Cardiovasc. Diabetol..

[40] Huang Y., Smith C.A., Chen G., Sharma B., Miner A.S., Barbee R.W., Ratz P.H. (2017). The AMP-dependent protein kinase (AMPK) activator A-769662 causes arterial relaxation by reducing cytosolic free calcium independently of an increase in AMPK phosphorylation.. Front. Pharmacol..

[41] Sung J.Y., Choi H.C. (2012). Metformin-induced AMP-activated protein kinase activation regulates phenylephrine-mediated contraction of rat aorta.. Biochem. Biophys. Res. Commun..

[42] Mori A., Ishikawa E., Amano T., Sakamoto K., Nakahara T. (2017). Anti-diabetic drug metformin dilates retinal blood vessels through activation of AMP-activated protein kinase in rats.. Eur. J. Pharmacol..

[43] Giacco F., Brownlee M. (2010). Oxidative stress and diabetic complications.. Circ. Res..

[44] Bellin C., de Wiza D.H., Wiernsperger N.F., Rösen P. (2006). Generation of reactive oxygen species by endothelial and smooth muscle cells: Influence of hyperglycemia and metformin.. Horm. Metab. Res..

[45] Ouslimani N., Peynet J., Bonnefont-Rousselot D., Thérond P., Legrand A., Beaudeux J-L. (2005). Metformin decreases intracellular production of reactive oxygen species in aortic endothelial cells.. Metabolism.

[46] Liu J., Aylor K.W., Chai W., Barrett E.J., Liu Z. (2022). Metformin prevents endothelial oxidative stress and microvascular insulin resistance during obesity development in male rats.. Am. J. Physiol. Endocrinol. Metab..

[47] Isoda K., Young J.L., Zirlik A., MacFarlane L.A., Tsuboi N., Gerdes N., Schönbeck U., Libby P. (2006). Metformin inhibits proinflammatory responses and nuclear factor-kappaB in human vascular wall cells.. Arterioscler. Thromb. Vasc. Biol..

[48] Arunachalam G., Samuel S.M., Marei I., Ding H., Triggle C.R. (2014). Metformin modulates hyperglycaemia-induced endothelial senescence and apoptosis through SIRT1.. Br. J. Pharmacol..

[49] Zheng Z., Chen H., Li J., Li T., Zheng B., Zheng Y., Jin H., He Y., Gu Q., Xu X. (2012). Sirtuin 1-mediated cellular metabolic memory of high glucose* via *the LKB1/AMPK/ROS pathway and therapeutic effects of metformin.. Diabetes.

[50] Guarente L. (2007). Sirtuins in aging and disease.. Cold Spring Harb. Symp. Quant. Biol..

[51] Sun C., Zhang F., Ge X., Yan T., Chen X., Shi X., Zhai Q. (2007). SIRT1 improves insulin sensitivity under insulin-resistant conditions by repressing PTP1B.. Cell Metab..

[52] Alcendor R.R., Gao S., Zhai P., Zablocki D., Holle E., Yu X., Tian B., Wagner T., Vatner S.F., Sadoshima J. (2007). Sirt1 regulates aging and resistance to oxidative stress in the heart.. Circ. Res..

[53] Potente M., Ghaeni L., Baldessari D., Mostoslavsky R., Rossig L., Dequiedt F., Haendeler J., Mione M., Dejana E., Alt F.W., Zeiher A.M., Dimmeler S. (2007). SIRT1 controls endothelial angiogenic functions during vascular growth.. Genes Dev..

[54] Elibol B., Kilic U. (2018). High levels of SIRT1 expression as a protective mechanism against disease-related conditions.. Front. Endocrinol. (Lausanne).

[55] Zu Y., Liu L., Lee M.Y., Xu C., Liang Y., Man R.Y., Vanhoutte P.M., Wang Y. (2010). SIRT1 promotes proliferation and prevents senescence through targeting LKB1 in primary porcine aortic endothelial cells.. Circ. Res..

[56] Mattagajasingh I., Kim C.S., Naqvi A., Yamamori T., Hoffman T.A., Jung S.B., DeRicco J., Kasuno K., Irani K. (2007). SIRT1 promotes endothelium-dependent vascular relaxation by activating endothelial nitric oxide synthase.. Proc. Natl. Acad. Sci. USA.

[57] Mohammed I., Hollenberg M.D., Ding H., Triggle C.R. (2021). A critical review of the evidence that metformin is a putative anti-aging drug that enhances healthspan and extends lifespan.. Front. Endocrinol. (Lausanne).

[58] Venu V.K.P., Saifeddine M., Mihara K., Faiza M., Gorobets E., Flewelling A.J., Derksen D.J., Hirota S.A., Marei I., Al-Majid D., Motahhary M., Ding H., Triggle C.R., Hollenberg M.D. (2021). MetfZormin prevents hyperglycemia-associated, oxidative stress-induced vascular endothelial dysfunction: Essential role for the orphan nuclear receptor human nuclear receptor 4A1 (Nur77).. Mol. Pharmacol..

[59] Zhan Y.Y., Chen Y., Zhang Q., Zhuang J.J., Tian M., Chen H.Z., Zhang L.R., Zhang H.K., He J.P., Wang W.J., Wu R., Wang Y., Shi C., Yang K., Li A.Z., Xin Y.Z., Li T.Y., Yang J.Y., Zheng Z.H., Yu C.D., Lin S.C., Chang C., Huang P.Q., Lin T., Wu Q. (2012). The orphan nuclear receptor Nur77 regulates LKB1 localization and activates AMPK.. Nat. Chem. Biol..

[60] Asahara T., Murohara T., Sullivan A., Silver M., van der Zee R., Li T., Witzenbichler B., Schatteman G., Isner J.M. (1997). Isolation of putative progenitor endothelial cells for angiogenesis.. Science.

[61] Dimmeler S., Zeiher A.M. (2004). Vascular repair by circulating endothelial progenitor cells: The missing link in atherosclerosis?. J. Mol. Med. (Berl.).

[62] Zhang M., Malik A.B., Rehman J. (2014). Endothelial progenitor cells and vascular repair.. Curr. Opin. Hematol..

[63] Berezin A. (2016). Epigenetically modified endothelial progenitor cells in heart failure.. J. Clin. Epigen..

[64] Berezin A., Berezin A. (2019). Endothelial progenitor cell dysfunction in diabetes mellitus.. New Target Risk Stratificat. Ther..

[65] Medina R.J., Barber C.L., Sabatier F., Dignat-George F., Melero-Martin J.M., Khosrotehrani K., Ohneda O., Randi A.M., Chan J.K.Y., Yamaguchi T., Van Hinsbergh V.W.M., Yoder M.C., Stitt A.W. (2017). Endothelial progenitors: A consensus statement on nomenclature.. Stem Cells Transl. Med..

[66] Yoon C.H., Hur J., Park K.W., Kim J.H., Lee C.S., Oh I.Y., Kim T.Y., Cho H.J., Kang H.J., Chae I.H., Yang H.K., Oh B.H., Park Y.B., Kim H.S. (2005). Synergistic neovascularization by mixed transplantation of early endothelial progenitor cells and late outgrowth endothelial cells: The role of angiogenic cytokines and matrix metalloproteinases.. Circulation.

[67] O’Neill T.J., Wamhoff B.R., Owens G.K., Skalak T.C. (2005). Mobilization of bone marrow-derived cells enhances the angiogenic response to hypoxia without transdifferentiation into endothelial cells.. Circ. Res..

[68] Zentilin L., Tafuro S., Zacchigna S., Arsic N., Pattarini L., Sinigaglia M., Giacca M. (2006). Bone marrow mononuclear cells are recruited to the sites of VEGF-induced neovascularization but are not incorporated into the newly formed vessels.. Blood.

[69] Fadini G.P., Miorin M., Facco M., Bonamico S., Baesso I., Grego F., Menegolo M., de Kreutzenberg S.V., Tiengo A., Agostini C., Avogaro A. (2005). Circulating endothelial progenitor cells are reduced in peripheral vascular complications of type 2 diabetes mellitus.. J. Am. Coll. Cardiol..

[70] Li M., Ho J.C., Lai K.W., Au K.K., Xu A., Cheung B.M., Lam K.S., Tse H.F. (2011). The decrement in circulating endothelial progenitor cells (EPCs) in type 2 diabetes is independent of the severity of the hypoadiponectemia.. Diabetes Metab. Res. Rev..

[71] Churdchomjan W., Kheolamai P., Manochantr S., Tapanadechopone P., Tantrawatpan C., U-Pratya Y., Issaragrisil S. (2010). Comparison of endothelial progenitor cell function in type 2 diabetes with good and poor glycemic control.. BMC Endocr. Disord..

[72] Yuan Q., Hu C.P., Gong Z.C., Bai Y.P., Liu S.Y., Li Y.J., Jiang J.L. (2015). Accelerated onset of senescence of endothelial progenitor cells in patients with type 2 diabetes mellitus: Role of dimethylarginine dimethylaminohydrolase 2 and asymmetric dimethylarginine.. Biochem. Biophys. Res. Commun..

[73] Reinhard H., Jacobsen P.K., Lajer M., Pedersen N., Billestrup N., Mandrup-Poulsen T., Parving H.H., Rossing P. (2010). Multifactorial treatment increases endothelial progenitor cells in patients with type 2 diabetes.. Diabetologia.

[74] Kränkel N., Adams V., Linke A., Gielen S., Erbs S., Lenk K., Schuler G., Hambrecht R. (2005). Hyperglycemia reduces survival and impairs function of circulating blood-derived progenitor cells.. Arterioscler. Thromb. Vasc. Biol..

[75] Tepper O.M., Galiano R.D., Capla J.M., Kalka C., Gagne P.J., Jacobowitz G.R., Levine J.P., Gurtner G.C. (2002). Human endothelial progenitor cells from type II diabetics exhibit impaired proliferation, adhesion, and incorporation into vascular structures.. Circulation.

[76] Chen Y.H., Lin S.J., Lin F.Y., Wu T.C., Tsao C.R., Huang P.H., Liu P.L., Chen Y.L., Chen J.W. (2007). High glucose impairs early and late endothelial progenitor cells by modifying nitric oxide-related but not oxidative stress-mediated mechanisms.. Diabetes.

[77] Zhang J., Zhang X., Li H., Cui X., Guan X., Tang K., Jin C., Cheng M. (2013). Hyperglycaemia exerts deleterious effects on late endothelial progenitor cell secretion actions.. Diab. Vasc. Dis. Res..

[78] Tura O., Skinner E.M., Barclay G.R., Samuel, K., Gallagher R.C., Brittan M., Hadoke P.W., Newby D.E., Turner M.L., Mills N.L. (2013). Late outgrowth endothelial cells resemble mature endothelial cells and are not derived from bone marrow.. Stem Cells.

[79] Ahmed F.W., Rider R., Glanville M., Narayanan K., Razvi S., Weaver J.U. (2016). Metformin improves circulating endothelial cells and endothelial progenitor cells in type 1 diabetes: MERIT study.. Cardiovasc. Diabetol..

[80] Chen L.L., Liao Y.F., Zeng T.S., Yu F., Li H.Q., Feng Y. (2010). Effects of metformin plus gliclazide compared with metformin alone on circulating endothelial progenitor cell in type 2 diabetic patients.. Endocrine.

[81] Dore F.J., Domingues C.C., Ahmadi N., Kundu N., Kropotova Y., Houston S., Rouphael C., Mammadova A., Witkin L., Khiyami A., Amdur R.L., Sen S. (2018). The synergistic effects of saxagliptin and metformin on CD34+ endothelial progenitor cells in early type 2 diabetes patients: A randomized clinical trial.. Cardiovasc. Diabetol..

[82] Yu J.W., Deng Y.P., Han X., Ren G.F., Cai J., Jiang G.J. (2016). Metformin improves the angiogenic functions of endothelial progenitor cells* via *activating AMPK/eNOS pathway in diabetic mice.. Cardiovasc. Diabetol..

[83] Han X., Tao Y., Deng Y., Yu J., Sun Y., Jiang G. (2017). Metformin accelerates wound healing in type 2 diabetic db/db mice.. Mol. Med. Rep..

[84] Dallaglio K., Bruno A., Cantelmo A.R., Esposito A.I., Ruggiero L., Orecchioni S., Calleri A., Bertolini F., Pfeffer U., Noonan D.M., Albini A. (2014). Paradoxic effects of metformin on endothelial cells and angiogenesis.. Carcinogenesis.

[85] Li W.D., Du X.L., Qian A.M., Hu N., Kong L.S., Wei S., Li C.L., Li X.Q. (2015). Metformin regulates differentiation of bone marrow-derived endothelial progenitor cells* via *multiple mechanisms.. Biochem. Biophys. Res. Commun..

[86] Li W.D., Li N.P., Song D.D., Rong J.J., Qian A.M., Li X.Q. (2017). Metformin inhibits endothelial progenitor cell migration by decreasing matrix metalloproteinases, MMP-2 and MMP-9,* via *the AMPK/mTOR/autophagy pathway.. Int. J. Mol. Med..

[87] Graham G.G., Punt J., Arora M., Day R.O., Doogue M.P., Duong J.K., Furlong T.J., Greenfield J.R., Greenup L.C., Kirkpatrick C.M., Ray J.E., Timmins P., Williams K.M. (2011). Clinical pharmacokinetics of metformin.. Clin. Pharmacokinet..

[88] Cabreiro F., Au C., Leung K.Y., Vergara-Irigaray N., Cochemé H.M., Noori T., Weinkove D., Schuster E., Greene N.D., Gems D. (2013). Metformin retards aging in C. elegans by altering microbial folate and methionine metabolism.. Cell.

[89] Chen J., Ou Y., Li Y., Hu S., Shao L.W., Liu Y. (2017). Metformin extends *C. elegans* lifespan through lysosomal pathway.. eLife.

[90] Espada L., Dakhovnik A., Chaudhari P., Martirosyan A., Miek L., Poliezhaieva T., Schaub Y., Nair A., Döring N., Rahnis N., Werz O., Koeberle A., Kirkpatrick J., Ori A., Ermolaeva M.A. (2020). Loss of metabolic plasticity underlies metformin toxicity in aged *Caenorhabditis elegans.*. Nat. Metab..

[91] Anisimov V.N., Berstein L.M., Popovich I.G., Zabezhinski M.A., Egormin P.A., Piskunova T.S., Semenchenko A.V., Tyndyk M.L., Yurova M.N., Kovalenko I.G., Poroshina T.E. (2011). If started early in life, metformin treatment increases life span and postpones tumors in female SHR mice.. Aging (Albany NY).

[92] Smith D.L., Elam C.F., Mattison J.A., Lane M.A., Roth G.S., Ingram D.K., Allison D.B. (2010). Metformin supplementation and life span in Fischer-344 rats.. J. Gerontol. A Biol. Sci. Med. Sci..

[93] Strong R., Miller R.A., Antebi A., Astle C.M., Bogue M., Denzel M.S., Fernandez E., Flurkey K., Hamilton K.L., Lamming D.W., Javors M.A., de Magalhães J.P., Martinez P.A., McCord J.M., Miller B.F., Müller M., Nelson J.F., Ndukum J., Rainger G.E., Richardson A., Sabatini D.M., Salmon A.B., Simpkins J.W., Steegenga W.T., Nadon N.L., Harrison D.E. (2016). Longer lifespan in male mice treated with a weakly estrogenic agonist, an antioxidant, an α-glucosidase inhibitor or a Nrf2-inducer.. Aging Cell.

[94] Glossmann H.H., Lutz O.M.D. (2019). Metformin and aging: A review.. Gerontology.

[95] Pedersen B.K., Saltin B. (2015). Exercise as medicine - evidence for prescribing exercise as therapy in 26 different chronic diseases.. Scand. J. Med. Sci. Sports.

[96] Leung F.P., Yung L.M., Laher I., Yao X., Chen Z.Y., Huang Y. (2008). Exercise, vascular wall and cardiovascular diseases: An update (Part 1).. Sports Med..

[97] Narkar V.A., Downes M., Yu R.T., Embler E., Wang Y.X., Banayo E., Mihaylova M.M., Nelson M.C., Zou Y., Juguilon H., Kang H., Shaw R.J., Evans R.M. (2008). AMPK and PPARdelta agonists are exercise mimetics.. Cell.

[98] Malin S.K., Braun B. (2016). Impact of metformin on exercise-induced metabolic adaptations to lower type 2 diabetes risk.. Exerc. Sport Sci. Rev..

[99] Konopka A.R., Laurin J.L., Schoenberg H.M., Reid J.J., Castor W.M., Wolff C.A., Musci R.V., Safairad O.D., Linden M.A., Biela L.M., Bailey S.M., Hamilton K.L., Miller B.F. (2019). Metformin inhibits mitochondrial adaptations to aerobic exercise training in older adults.. Aging Cell.

[100] Terada T., Boulé N.G. (2019). Does metformin therapy influence the effects of intensive lifestyle intervention? Exploring the interaction between first line therapies in the Look AHEAD trial.. Metabolism.

[101] Walton R.G., Dungan C.M., Long D.E., Tuggle S.C., Kosmac K., Peck B.D., Bush H.M., Villasante Tezanos A.G., McGwin G., Windham S.T., Ovalle F., Bamman M.M., Kern P.A., Peterson C.A. (2019). Metformin blunts muscle hypertrophy in response to progressive resistance exercise training in older adults: A randomized, double-blind, placebo-controlled, multicenter trial: The MASTERS trial.. Aging Cell.

[102] Malin S.K., Gerber R., Chipkin S.R., Braun B. (2012). Independent and combined effects of exercise training and metformin on insulin sensitivity in individuals with prediabetes.. Diabetes Care.

[103] Gebrie D., Getnet D., Manyazewal T. (2021). Cardiovascular safety and efficacy of metformin-SGLT2i* versus *metformin-sulfonylureas in type 2 diabetes: Systematic review and meta-analysis of randomized controlled trials.. Sci. Rep..

[104] Zaccardi F., Kloecker D.E., Buse J.B., Mathieu C., Khunti K., Davies M.J. (2021). Use of metformin and cardiovascular effects of new classes of glucose-lowering agents: A meta-analysis of cardiovascular outcome trials in type 2 diabetes.. Diabetes Care.

[105] Masson W., Lavalle-Cobo A., Lobo M., Masson G., Molinero G. (2021). Novel antidiabetic drugs and risk of cardiovascular events in patients without baseline metformin use: A meta-analysis.. Eur. J. Prev. Cardiol..

[106] Lunder M., Janić M., Japelj M., Juretič A., Janež A., Šabovič M. (2018). Empagliflozin on top of metformin treatment improves arterial function in patients with type 1 diabetes mellitus.. Cardiovasc. Diabetol..

[107] Singh A.K., Singh R. (2019). Heart failure hospitalization with SGLT-2 inhibitors: A systematic review and meta-analysis of randomized controlled and observational studies.. Expert Rev. Clin. Pharmacol..

[108] Salvatore T., Galiero R., Caturano A., Vetrano E., Rinaldi L., Coviello F., Di Martino A., Albanese G., Marfella R., Sardu C., Sasso F.C. (2021). Effects of metformin in heart failure: From pathophysiological rationale to clinical evidence.. Biomolecules.

[109] Schernthaner G. (2021). Can glucose-lowering drugs affect the prognosis of COVID-19 in patients with type 2 diabetes?. Lancet Diabetes Endocrinol..

[110] Wiggers H., Køber L., Gislason G., Schou M., Poulsen M.K., Vraa S., Nielsen O.W., Bruun N.E., Nørrelund H., Hollingdal M., Barasa A., Bøttcher M., Dodt K., Hansen V.B., Nielsen G., Knudsen A.S., Lomholdt J., Mikkelsen K.V., Jonczy B., Brønnum-Schou J., Poenaru M.P., Abdulla J., Raymond I., Mahboubi K., Sillesen K., Serup-Hansen K., Madsen J.S., Kristensen S.L., Larsen A.H., Bøtker H.E., Torp-Petersen C., Eiskjær H., Møller J., Hassager C., Steffensen F.H., Bibby B.M., Refsgaard J., Høfsten D.E., Mellemkjær S., Gustafsson F. (2021). The DANish randomized, double-blind, placebo controlled trial in patients with chronic HEART failure (DANHEART): A 2 × 2 factorial trial of hydralazine-isosorbide dinitrate in patients with chronic heart failure (H-HeFT) and metformin in patients with chronic heart failure and diabetes or prediabetes (Met-HeFT).. Am. Heart J..

[111] Farmer R.E., Beard I., Raza S.I., Gollop N.D., Patel N., Tebboth A., McGovern A.P., Kanumilli N., Ternouth A. (2021). Prescribing in type 2 diabetes patients with and without cardiovascular disease history: A descriptive analysis in the UK CPRD.. Clin. Ther..

[112] Hemmingsen B., Schroll J.B., Wetterslev J., Gluud C., Vaag A., Sonne D.P., Lundstrøm L.H., Almdal T. (2014). Sulfonylurea* versus *metformin monotherapy in patients with type 2 diabetes: A Cochrane systematic review and meta-analysis of randomized clinical trials and trial sequential analysis.. CMAJ Open.

[113] Turner R.C., Cull C.A., Frighi V., Holman R.R., UK Prospective Diabetes Study (UKPDS) Group (1999). Glycemic control with diet, sulfonylurea, metformin, or insulin in patients with type 2 diabetes mellitus: Progressive requirement for multiple therapies (UKPDS 49).. JAMA.

[114] De Broe M.E., Jouret F. (2020). Does metformin do more benefit or harm in chronic kidney disease patients?. Kidney Int..

[115] Hanna R.M., Rhee C.M., Kalantar-Zadeh K. (2020). Metformin in chronic kidney disease: A strong dose of caution.. Kidney Int..

[116] Corremans R., Vervaet B.A., D’Haese P.C., Neven E., Verhulst A. (2018). Metformin: A candidate drug for renal diseases.. Int. J. Mol. Sci..

[117] Lalau J-D., Kajbaf F., Bennis Y., Hurtel-Lemaire A-S., Belpaire F., De Broe M.E. (2018). Metformin treatment in patients with type 2 diabetes and chronic kidney disease stages 3A, 3B, or 4.. Diabetes Care.

[118] Salvatore T., Pafundi P.C., Marfella R., Sardu C., Rinaldi L., Monaco L., Ricozzi C., Imbriani S., Nevola R., Adinolfi L.E., Sasso F.C. (2019). Metformin lactic acidosis: Should we still be afraid?. Diabetes Res. Clin. Pract..

[119] Hung A.M., Roumie C.L., Greevy R.A., Liu X., Grijalva C.G., Murff H.J., Griffin M.R. (2013). Kidney function decline in metformin* versus *sulfonylurea initiators: Assessment of time-dependent contribution of weight, blood pressure, and glycemic control.. Pharmacoepidemiol. Drug Saf..

[120] Crowley M.J., Diamantidis C.J., McDuffie J.R., Cameron C.B., Stanifer J.W., Mock C.K., Wang X., Tang S., Nagi A., Kosinski A.S., Williams J.W. (2017). Clinical outcomes of metformin use in populations with chronic kidney disease, congestive heart failure, or chronic liver disease: A systematic review.. Ann. Intern. Med..

[121] Stephen J., Anderson-Haag T.L., Gustafson S., Snyder J.J., Kasiske B.L., Israni A.K. (2014). Metformin use in kidney transplant recipients in the United States: An observational study.. Am. J. Nephrol..

[122] Lin C-X., Li Y., Liang S., Tao J., Zhang L-S., Su Y-F., Huang Y-X., Zhao Z-K., Liu S-Y., Zheng J-M. (2019). Metformin attenuates cyclosporine A-induced renal fibrosis in rats.. Transplantation.

[123] Neven E., Vervaet B., Brand K., Gottwald-Hostalek U., Opdebeeck B., De Maré A., Verhulst A., Lalau J-D., Kamel S., De Broe M.E., D’Haese P.C. (2018). Metformin prevents the development of severe chronic kidney disease and its associated mineral and bone disorder.. Kidney Int..

[124] Satriano J., Sharma K., Blantz R.C., Deng A. (2013). Induction of AMPK activity corrects early pathophysiological alterations in the subtotal nephrectomy model of chronic kidney disease.. Am. J. Physiol. Renal Physiol..

[125] Mohamad H.E., Asker M.E., Keshawy M.M., Abdel Aal S.M., Mahmoud Y.K. (2020). Infliximab ameliorates tumor necrosis factor-alpha exacerbated renal insulin resistance induced in rats by regulating insulin signaling pathway.. Eur. J. Pharmacol..

[126] Pan Q., Lu X., Zhao C., Liao S., Chen X., Guo F., Yang C., Liu H.F. (2020). Metformin: The updated protective property in kidney disease.. Aging (Albany NY).

[127] Song A., Zhang C., Meng X. (2021). Mechanism and application of metformin in kidney diseases: An update.. Biomed. Pharmacother..

[128] Chen Y., Yang D., Cheng B., Chen J., Peng A., Yang C., Liu C., Xiong M., Deng A., Zhang Y., Zheng L., Huang K., Clinical characteristics and outcomes of patients with diabetes and COVID-19 in association with glucose-lowering medication (2020). Clinical characteristics and outcomes of patients with diabetes and COVID-19 in association with glucose-lowering medication.. Diabetes Care.

[129] Fadini G.P., Morieri M.L., Longato E., Avogaro A. (2020). Prevalence and impact of diabetes among people infected with SARS-CoV-2.. J. Endocrinol. Invest..

[130] Guan W.J., Ni Z.Y., Hu Y., Liang W.H., Ou C.Q., He J.X., Liu L., Shan H., Lei C.L., Hui D.S.C., Du B., Li L.J., Zeng G., Yuen K-Y., Chen R.C., Tang C.L., Wang T., Chen P.Y., Xiang J., Li S.Y., Wang J.L., Liang Z.J., Peng Y.X., Wei L., Liu Y., Hu Y.H., Peng P., Wang J.M., Liu J.Y., Chen Z., Li G., Zheng Z.J., Qiu S.Q., Luo J., Ye C.J., Zhu S.Y., Zhong N.S., China Medical Treatment Expert Group for Covid-19 (2020). 2019 in China.. N. Engl. J. Med..

[131] Wu C., Chen X., Cai Y., Xia J., Zhou X., Xu S., Huang H., Zhang L., Zhou X., Du C., Zhang Y., Song J., Wang S., Chao Y., Yang Z., Xu J., Zhou X., Chen D., Xiong W., Xu L., Zhou F., Jiang J., Bai C., Zheng J., Song Y., Risk Factors Associated With Acute Respiratory Distress Syndrome and Death in Patients With Coronavirus Disease (2020). 2019 Pneumonia in Wuhan, China.. JAMA Intern. Med..

[132] Hamer M., Gale C.R., Kivimäki M., Batty G.D. (2020). Overweight, obesity, and risk of hospitalization for COVID-19: A community-based cohort study of adults in the United Kingdom.. Proc. Natl. Acad. Sci. USA.

[133] Drucker D.J. (2021). Diabetes, obesity, metabolism, and SARS-CoV-2 infection: The end of the beginning.. Cell Metab..

[134] Coppelli A., Giannarelli R., Aragona M., Penno G., Falcone M., Tiseo G., Ghiadoni L., Barbieri G., Monzani F., Virdis A., Menichetti F., Del Prato S., Pisa COVID-19 Study Group (2020). Hyperglycemia at hospital admission is associated with severity of the prognosis in patients hospitalized for COVID-19: The pisa COVID-19 study.. Diabetes Care.

[135] Mamtani M., Kulkarni H., Bihari S., Prakash S., Chavan S., Huckson S., Pilcher D. (2020). Degree of hyperglycemia independently associates with hospital mortality and length of stay in critically ill, nondiabetic patients: Results from the ANZICS CORE binational registry.. J. Crit. Care.

[136] Zhu L., She Z.G., Cheng X., Qin J.J., Zhang X.J., Cai J., Lei F., Wang H., Xie J., Wang W., Li H., Zhang P., Song X., Chen X., Xiang M., Zhang C., Bai L., Xiang D., Chen M.M., Liu Y., Yan Y., Liu M., Mao W., Zou J., Liu L., Chen G., Luo P., Xiao B., Zhang C., Zhang Z., Lu Z., Wang J., Lu H., Xia X., Wang D., Liao X., Peng G., Ye P., Yang J., Yuan Y., Huang X., Guo J., Zhang B.H., Li H. (2020). Association of Blood Glucose Control and Outcomes in Patients with COVID-19 and Pre-existing Type 2 Diabetes.. Cell Metab..

[137] Ceriello A. (2020). Hyperglycemia and the worse prognosis of COVID-19. Why a fast blood glucose control should be mandatory.. Diabetes Res. Clin. Pract..

[138] Reiterer M., Rajan M., Gómez-Banoy N., Lau J.D., Gomez-Escobar L.G., Ma L., Gilani A., Alvarez-Mulett S., Sholle E.T., Chandar V., Bram Y., Hoffman K., Bhardwaj P., Piloco P., Rubio-Navarro A., Uhl S., Carrau L., Houhgton S., Redmond D., Shukla A.P., Goyal P., Brown K.A., tenOever B.R., Alonso L.C., Schwartz R.E., Schenck E.J., Safford M.M., Lo J.C. (2021). Hyperglycemia in acute COVID-19 is characterized by insulin resistance and adipose tissue infectivity by SARS-CoV-2.. Cell Metab..

[139] Kim N.H., Kim K.J., Choi J., Kim S.G. (2022). Metabolically unhealthy individuals, either with obesity or not, have a higher risk of critical coronavirus disease 2019 outcomes than metabolically healthy individuals without obesity.. Metabolism.

[140] Sanoudou D., Hill M.A., Belanger M.J., Arao K., Mantzoros C.S. (2022). Editorial: Obesity, metabolic phenotypes and COVID-19.. Metabolism.

[141] Bramante C.T., Ingraham N.E., Murray T.A., Marmor S., Hovertsen S., Gronski J., McNeil C., Feng R., Guzman G., Abdelwahab N., King S., Tamariz L., Meehan T., Pendleton K.M., Benson B., Vojta D., Tignanelli C.J. (2021). Metformin and risk of mortality in patients hospitalised with COVID-19: A retrospective cohort analysis.. Lancet Healthy Longev..

[142] Lukito A.A., Pranata R., Henrina J., Lim M.A., Lawrensia S., Suastika K. (2020). The Effect of Metformin Consumption on Mortality in Hospitalized COVID-19 patients: A systematic review and meta-analysis.. Diabetes Metab. Syndr..

[143] Crouse A., Grimes T., Li P., Might M., Ovalle F., Shalev A. (2020). Metformin use is associated with reduced mortality in a diverse population with COVID-19 and diabetes.. medRxiv.

[144] Wargny M., Potier L., Gourdy P., Pichelin M., Amadou C., Benhamou P.Y., Bonnet J.B., Bordier L., Bourron O., Chaumeil C., Chevalier N., Darmon P., Delenne B., Demarsy D., Dumas M., Dupuy O., Flaus-Furmaniuk A., Gautier J.F., Guedj A.M., Jeandidier N., Larger E., Le Berre J.P., Lungo M., Montanier N., Moulin P., Plat F., Rigalleau V., Robert R., Seret-Bégué D., Sérusclat P., Smati S., Thébaut J.F., Tramunt B., Vatier C., Velayoudom F.L., Vergès B., Winiszewski P., Zabulon A., Gourraud P.A., Roussel R., Cariou B., Hadjadj S., CORONADO investigators (2021). Predictors of hospital discharge and mortality in patients with diabetes and COVID-19: Updated results from the nationwide CORONADO study.. Diabetologia.

[145] Zangiabadian M., Nejadghaderi S.A., Zahmatkesh M.M., Hajikhani B., Mirsaeidi M., Nasiri M.J. (2021). The efficacy and potential mechanisms of metformin in the treatment of COVID-19 in the diabetics: A systematic review.. Front. Endocrinol. (Lausanne).

[146] Evia-Viscarra M.L., Rodea-Montero E.R., Apolinar-Jiménez E., Muñoz-Noriega N., García-Morales L.M., Leaños-Pérez C., Figueroa-Barrón M., Sánchez-Fierros D., Reyes-García J.G. (2012). The effects of metformin on inflammatory mediators in obese adolescents with insulin resistance: Controlled randomized clinical trial.. J. Pediatr. Endocrinol. Metab..

[147] Cameron A.R., Morrison V.L., Levin D., Mohan M., Forteath C., Beall C., McNeilly A.D., Balfour D.J., Savinko T., Wong A.K., Viollet B., Sakamoto K., Fagerholm S.C., Foretz M., Lang C.C., Rena G. (2016). Anti-inflammatory effects of metformin irrespective of diabetes status.. Circ. Res..

[148] Zahorec R. (2021). Neutrophil-to-lymphocyte ratio, past, present and future perspectives.. Bratisl. Lek Listy.

[149] Gordon D.E., Jang G.M., Bouhaddou M., Xu J., Obernier K., White K.M., O’Meara M.J., Rezelj V.V., Guo J.Z., Swaney D.L., Tummino T.A., Hüttenhain R., Kaake R.M., Richards A.L., Tutuncuoglu B., Foussard H., Batra J., Haas K., Modak M., Kim M., Haas P., Polacco B.J., Braberg H., Fabius J.M., Eckhardt M., Soucheray M., Bennett M.J., Cakir M., McGregor M.J., Li Q., Meyer B., Roesch F., Vallet T., Mac Kain A., Miorin L., Moreno E., Naing Z.Z.C., Zhou Y., Peng S., Shi Y., Zhang Z., Shen W., Kirby I.T., Melnyk J.E., Chorba J.S., Lou K., Dai S.A., Barrio-Hernandez I., Memon D., Hernandez-Armenta C., Lyu J., Mathy C.J.P., Perica T., Pilla K.B., Ganesan S.J., Saltzberg D.J., Rakesh R., Liu X., Rosenthal S.B., Calviello L., Venkataramanan S., Liboy-Lugo J., Lin Y., Huang X-P., Liu Y., Wankowicz S.A., Bohn M., Safari M., Ugur F.S., Koh C., Savar N.S., Tran Q.D., Shengjuler D., Fletcher S.J., O’Neal M.C., Cai Y., Chang J.C.J., Broadhurst D.J., Klippsten S., Sharp P.P., Wenzell N.A., Kuzuoglu-Ozturk D., Wang H-Y., Trenker R., Young J.M., Cavero D.A., Hiatt J., Roth T.L., Rathore U., Subramanian A., Noack J., Hubert M., Stroud R.M., Frankel A.D., Rosenberg O.S., Verba K.A., Agard D.A., Ott M., Emerman M., Jura N., von Zastrow M., Verdin E., Ashworth A., Schwartz O., d’Enfert C., Mukherjee S., Jacobson M., Malik H.S., Fujimori D.G., Ideker T., Craik C.S., Floor S.N., Fraser J.S., Gross J.D., Sali A., Roth B.L., Ruggero D., Taunton J., Kortemme T., Beltrao P., Vignuzzi M., García-Sastre A., Shokat K.M., Shoichet B.K., Krogan N.J. (2020). A SARS-CoV-2 protein interaction map reveals targets for drug repurposing.. Nature.

[150] Kindrachuk J., Ork B., Hart B.J., Mazur S., Holbrook M.R., Frieman M.B., Traynor D., Johnson R.F., Dyall J., Kuhn J.H., Olinger G.G., Hensley L.E., Jahrling P.B. (2015). Antiviral potential of ERK/MAPK and PI3K/AKT/mTOR signaling modulation for Middle East respiratory syndrome coronavirus infection as identified by temporal kinome analysis.. Antimicrob. Agents Chemother..

[151] Zhang J., Dong J., Martin M., He M., Gongol B., Marin T.L., Chen L., Shi X., Yin Y., Shang F., Wu Y., Huang H.Y., Zhang J., Zhang Y., Kang J., Moya E.A., Huang H.D., Powell F.L., Chen Z., Thistlethwaite P.A., Yuan Z.Y., Shyy J.Y. (2018). AMP-activated protein kinase phosphorylation of angiotensin-converting enzyme 2 in endothelium mitigates pulmonary hypertension.. Am. J. Respir. Crit. Care Med..

[152] Kamyshnyi O., Matskevych V., Lenchuk T., Strilbytska O., Storey K., Lushchak O. (2021). Metformin to decrease COVID-19 severity and mortality: Molecular mechanisms and therapeutic potential.. Biomed. Pharmacother..

[153] McFadyen J.D., Stevens H., Peter K., The Emerging Threat of (2020). The emerging threat of (micro)thrombosis in COVID-19 and its therapeutic implications.. Circ. Res..

[154] Varga Z., Flammer A.J., Steiger P., Haberecker M., Andermatt R., Zinkernagel A.S., Mehra M.R., Schuepbach R.A., Ruschitzka F., Moch H. (2020). Endothelial cell infection and endotheliitis in COVID-19.. Lancet.

[155] Katsiki N., Ferrannini E. (2020). Anti-inflammatory properties of antidiabetic drugs: A “promised land” in the COVID-19 era?. J. Diabetes Complications.

[156] Pollack R.M., Donath M.Y., LeRoith D., Leibowitz G. (2016). Anti-inflammatory agents in the treatment of diabetes and its vascular complications.. Diabetes Care.

[157] Pérez-Belmonte L.M., Torres-Peña J.D., López-Carmona M.D., Ayala-Gutiérrez M.M., Fuentes-Jiménez F., Huerta L.J., Muñoz J.A., Rubio-Rivas M., Madrazo M., Garcia M.G., Montes B.V., Sola J.F., Ena J., Ferrer R.G., Pérez C.M., Ripper C.J., Lecumberri J.J.N., Acedo I.E.A., Canteli S.P., Cosío S.F., Martínez F.A., Rodríguez B.C., Pérez-Martínez P., Ramos-Rincón J.M., Gómez-Huelgas R., SEMI-COVID-19 Network (2020). Mortality and other adverse outcomes in patients with type 2 diabetes mellitus admitted for COVID-19 in association with glucose-lowering drugs: A nationwide cohort study.. BMC Med..

[158] Cariou B., Hadjadj S., Wargny M., Pichelin M., Al-Salameh A., Allix I., Amadou C., Arnault G., Baudoux F., Bauduceau B., Borot S., Bourgeon-Ghittori M., Bourron O., Boutoille D., Cazenave-Roblot F., Chaumeil C., Cosson E., Coudol S., Darmon P., Disse E., Ducet-Boiffard A., Gaborit B., Joubert M., Kerlan V., Laviolle B., Marchand L., Meyer L., Potier L., Prevost G., Riveline J-P., Robert R., Saulnier P-J., Sultan A., Thébaut J-F., Thivolet C., Tramunt B., Vatier C., Roussel R., Gautier J-F., Gourdy P., CORONADO investigators (2020). Phenotypic characteristics and prognosis of inpatients with COVID-19 and diabetes: The CORONADO study.. Diabetologia.

[159] Khunti K., Knighton P., Zaccardi F., Bakhai C., Barron E., Holman N., Kar P., Meace C., Sattar N., Sharp S., Wareham N.J., Weaver A., Woch E., Young B., Valabhji J. (2021). Prescription of glucose-lowering therapies and risk of COVID-19 mortality in people with type 2 diabetes: A nationwide observational study in England.. Lancet Diabetes Endocrinol..

[160] Hadjadj S., Wargny M. (2021). Glucose-lowering treatments and COVID-19 mortality in T2DM.. Nat. Rev. Endocrinol..

[161] Santos A., Magro D.O., Evangelista-Poderoso R., Saad M.J.A. (2021). Diabetes, obesity, and insulin resistance in COVID-19: Molecular interrelationship and therapeutic implications.. Diabetol. Metab. Syndr..

[162] Stevens A., Hamel J.F., Toure A., Hadjadj S., Boels D. (2019). Metformin overdose: A serious iatrogenic complication—Western france poison control centre data analysis.. Basic Clin. Pharmacol. Toxicol..

